# Theory of Electron Correlation in Disordered Crystals

**DOI:** 10.3390/ma15030739

**Published:** 2022-01-19

**Authors:** Stanislav P. Repetsky, Iryna G. Vyshyvana, Sergei P. Kruchinin, Stefano Bellucci

**Affiliations:** 1 G. V. Kurdyumov Institute for Metal Physics of the NAS of Ukraine, 03142 Kyiv, Ukraine; srepetsky0208@gmail.com; 2 Department of Physical and Mathematical Sciences, National University of Kyiv-Mohyla Academy, 04070 Kyiv, Ukraine; 3 Institute of High Technologies, Taras Shevchenko Kyiv National University, 01033 Kyiv, Ukraine; i.vyshyvana@gmail.com; 4 Bogolyubov Institute for Theoretical Physics, NASU, 03143 Kyiv, Ukraine; sergeikruchinin@yahoo.com; 5 INFN-Laboratori Nazionali di Frascati, Via E. Fermi, 54, 00044 Frascati, Italy

**Keywords:** electronic spectrum, electrical conductivity, disordered crystals, the Hamiltonian of electrons and phonons, Green’s functions, the temperature Green’s functions, diagram technics, the mass operator of the Green’s function, density of states, free energy

## Abstract

This paper presents a new method of describing the electronic spectrum and electrical conductivity of disordered crystals based on the Hamiltonian of electrons and phonons. Electronic states of a system are described by the tight-binding model. Expressions for Green’s functions and electrical conductivity are derived using the diagram method. Equations are obtained for the vertex parts of the mass operators of the electron–electron and electron–phonon interactions. A system of exact equations is obtained for the spectrum of elementary excitations in a crystal. This makes it possible to perform numerical calculations of the energy spectrum and to predict the properties of the system with a predetermined accuracy. In contrast to other approaches, in which electron correlations are taken into account only in the limiting cases of an infinitely large and infinitesimal electron density, in this method, electron correlations are described in the general case of an arbitrary density. The cluster expansion is obtained for the density of states and electrical conductivity of disordered systems. We show that the contribution of the electron scattering processes to clusters is decreasing, along with increasing the number of sites in the cluster, which depends on a small parameter.

## 1. Introduction

Advances in the description of disordered systems are mainly due to the development of the pseudopotential method [1]. However, due to the nonlocal nature of the pseudopotential, there is a problem of portability of the pseudopotential. It is impossible to use nuclear potentials, determined by the properties of some systems, in order to describe other systems. The use of the theory of Vanderbilt ultra-soft potentials [2,3] and the method of projector-augmented waves proposed by Blochl [4,5], allowed for achieving fundamental progress in investigating the electronic structure and the properties of the system. In the augmented-wave projector method, the wave function of the valence states of an electron (all-electron orbital) is expressed in terms of a pseudo-wave function. The pseudo wave function is expanded in a series of pseudo partial wave functions. The wave function is expanded in a series of partial wave functions with the same coefficients as in the expression for the pseudo wave function. Partial wave functions are described by the Schrödinger equation for non-interacting atoms. The expression for the pseudo Hamiltonian, as an equation for the pseudo wave function, is derived by minimizing the full energy functional. This approach was further developed through the use of the generalized gradient approximation proposed in [6,7,8,9,10]. The paper [10] describes the application of this method for calculating the electronic structure of crystals and molecules using the VASP and GAUSSIAN software packages, respectively.

It should be noted that in [11,12,13,14,15,16,17,18,19], the crystals electronic structure was carried out, including the Coulomb long-range interaction between electrons of different sites on the crystal lattice, thanks to a method based on the tight-binding model [20,21] and the functional density theory. However, such methods are suitable only for describing crystals characterized by ideal ordering. In disordered crystals, effects associated with localized electronic states occur. These effects cannot be described in a model where the crystal is treated as an ideal one.

Calculations of the electronic structure of an alloy are based on using the self-consistent method of the Korringa–Kohn–Rostoker-coherent potential approximations are made in work [22,23,24]. In [25], a virtual crystal approximation was proposed to study the properties of alloys by the density functional method. This approach is applied in the Vanderbilt ultra-soft pseudopotential scheme to predict the properties of Pb(Zr_0.5_Ti_0.5_)O_3_ alloys in its paraelectric and ferroelectric phases.

In our work [26], we present a new method of describing the electronic spectrum and electrical conductivity of disordered crystals based on the Hamiltonian of electrons and phonons. Electronic states of a system are described by the tight-binding model. Calculations of two-time Green’s functions are based on temperature Green’s functions [26]. This uses a known relation between spectral representation for two-time and temperature Green’s function [27]. The calculation of the temperature Green’s functions of disordered crystal based on diagram technics are analogous to the diagram technique for a homogeneous system [27]. A system of exact equations is obtained for the spectrum of elementary excitations in a crystal. This makes it possible to perform numerical calculations of the energy spectrum and to predict the properties of the system with a predetermined accuracy.

## 2. Hamiltonian of an Electron–Phonon System for a Disordered Crystal

The Hamiltonian of the disordered system (alloy, disordered semiconductor, and disordered dielectric) consists of the sum of the Hamiltonian of electrons in the nucleus field, the Hamiltonian of electron–electron interaction, and the Hamilton of nucleus. The motion of the ion subsystem is reduced to nucleus oscillations near the equilibrium position under the influence of the nuclei interaction force, and their indirect interaction through electrons. In the Wannier representation, the system Hamiltonian is as follows [26]:

(1) $$H=H_{0}+H_{\mathrm{int}}$$

where zero-order Hamiltonian

(2) $$H_{0}=H_{e}^{\left(0\right)}+H_{ph}^{\left(0\right)}$$

consists of the Hamiltonian of the electrons in the field of the cores of the atom’s ideal ordered crystal

(3) $$H_{e}^{\left(0\right)}=\sum_{\begin{array}{c} ni\gamma \\ n^{\prime }i^{\prime }\gamma^{\prime } \end{array}}h_{ni\gamma ,n^{\prime }i^{\prime }\gamma^{\prime }}^{\left(0\right)}a_{ni\gamma }^{+}a_{n^{\prime }i^{\prime }\gamma^{\prime }}$$

and the harmonic phonon Hamiltonian for the motion of the cores of the atom’s ideal ordered crystal

(4) $$H_{ph}^{\left(0\right)}=\sum_{ni\alpha }\frac{P_{ni\alpha }^{2}}{2M_{i}}+\frac{1}{2}\sum_{\begin{array}{c} ni\alpha \\ n^{\prime }i^{\prime }\alpha^{\prime } \end{array}}\Phi_{ni\alpha ,n^{\prime }i^{\prime }\alpha^{\prime }}^{\left(0\right)}u_{ni\alpha }u_{n^{\prime }i^{\prime }\alpha^{\prime }}$$

Symbol *n* denotes the number of a unit cell, *i* denotes the number of a node in a unit cell, and γ denotes all of the other quantum numbers for the orbital, including spin. The symbol *h*^(0)^ denotes the “hopping integral” that connects the respective orbitals. For the phonon Hamiltonian, α is a spatial direction (*x*, *y*, or *z*), $P_{ni\alpha }$ is the core momentum, $M_{i}$ is the mass of the core, $u_{ni\alpha }$ is the deviation of the core from the equilibrium position of the lattice site, and $\Phi_{ni\alpha ,n^{\prime }i^{\prime }\alpha^{\prime }}^{\left(0\right)}$ is the corresponding spring-constant matrix.

The interaction Hamiltonian in Equation (1) is the perturbation of the system due to all of the effects we will be including. It is composed of six pieces:

(5) $$H_{\mathrm{int}}=\delta \Phi +H_{ec}+H_{eph}+H_{ee}+H_{phc}+H_{phph}$$

δΦ is the modification of the core–core Coulomb interaction due to the disordered atoms added to the system; it is the difference between the original core–core repulsion Hamiltonian and the new one. The electronic Hamiltonian is modified by the term

(6) $$H_{ec}=\sum_{\begin{array}{c} ni\gamma \\ n^{\prime }i^{\prime }\gamma^{\prime } \end{array}}w_{ni\gamma ,n^{\prime }i^{\prime }\gamma^{\prime }}a_{ni\gamma }^{+}a_{n^{\prime }i^{\prime }\gamma^{\prime }}$$

which is the difference between the new hopping Hamiltonian and the original periodic one. The electron–phonon interaction is given by

(7) $$H_{eph}=\sum_{\begin{array}{c} ni\gamma \\ n^{\prime }i^{\prime }\gamma^{\prime } \end{array}}v_{\;ni\gamma ,n^{\prime }i^{\prime }\gamma^{\prime }}^{\prime }a_{ni\gamma }^{+}a_{n^{\prime }i^{\prime }\gamma^{\prime }}$$

It is described in more detail below. The Hamiltonian of the Coulomb interaction between electrons is given by the term

(8) $$\begin{array}{l} H_{ee}=\frac{1}{2}\sum_{\begin{array}{l} n_{1},n_{2},\\ n_{3},n_{4} \end{array}}v_{n_{3},n_{4}}^{\left(2\right)n_{1},n_{2}}a_{n_{1}}^{+}a_{n_{2}}^{+}a_{n_{3}}a_{n_{4}},\\ n=\left(ni\gamma \right). \end{array}$$

The modification of the interaction of the phonons with the cores caused by the disordering of the atoms is given by

(9) $$\begin{array}{l} H_{phc}=\frac{1}{2}\sum_{\begin{array}{c} ni\alpha \\ n^{\prime }i^{\prime }\alpha^{\prime } \end{array}}\Delta M_{ni\alpha ,n^{\prime }i^{\prime }\alpha^{\prime }}^{-1}P_{ni\alpha }P_{n^{\prime }i^{\prime }\alpha^{\prime }}+\\ +\frac{1}{2}\sum_{\begin{array}{c} ni\alpha \\ n^{\prime }i^{\prime }\alpha^{\prime } \end{array}}\Delta \Phi_{ni\alpha ,n^{\prime }i^{\prime }\alpha^{\prime }}u_{ni\alpha }u_{n^{\prime }i^{\prime }\alpha^{\prime }}, \end{array}$$

where

(10) $$\Delta M_{ni\alpha ,n^{\prime }i^{\prime }\alpha^{\prime }}^{-1}=\left(\frac{1}{M_{ni^{\prime }}}-\frac{1}{M_{i}}\right)\delta_{nn^{\prime }}\delta_{ii^{\prime }}\delta_{\alpha \alpha^{\prime }}$$

$\Delta \Phi_{ni\alpha ,n^{\prime }i^{\prime }\alpha^{\prime }}=\Phi_{ni\alpha ,n^{\prime }i^{\prime }\alpha^{\prime }}-\Phi_{ni\alpha ,n^{\prime }i^{\prime }\alpha^{\prime }}^{\left(0\right)}$, and $M_{ni}$ and $M_{i}$ are the masses of the atoms at site (ni) for the disordered and ordered alloy, respectively.

We also include the cubic anharmonic potential terms for the phonons (under the assumption that they remain small and can be treated perturbatively via

(11) $$\begin{array}{l} H_{phph}=\frac{1}{3!}\sum_{\begin{array}{l} ni\alpha \\ n^{\prime }i^{\prime }\alpha^{\prime }\\ n^{\prime \prime }i^{\prime \prime }\alpha^{\prime \prime } \end{array}}\Phi_{ni\alpha ,n^{\prime }i^{\prime }\alpha^{\prime },n^{\prime \prime }i^{\prime \prime }\alpha^{\prime \prime }}^{\left(0\right)}u_{ni\alpha }\times \\ \times u_{n^{\prime }i^{\prime }\alpha^{\prime }}u_{n^{\prime \prime }i^{\prime \prime }\alpha^{\prime \prime }}. \end{array}$$

The operators $a_{ni\gamma }^{+}$ and $a_{ni\gamma }^{}$ create and destroy electrons in the state described by Vane’s function $\phi_{ni\gamma }\left(\xi \right)=\langle \xi |ni\gamma \rangle$, where $\xi =\left(r,\sigma^{\prime }\right)$ are the spatial and *z*-components of the spin coordinates of the wave function.

To construct the Wannier functions, we use analytical expressions for the wave functions of an electron in the field of atomic nuclei of type λ localized at the lattice sites (ni) of an ideally ordered crystal:

(12) $$\begin{array}{l} \psi_{ni\delta }\left(r-r_{ni}\right)=R_{\tilde{\varepsilon }l}\left(\left|r-r_{ni}\right|\right)Y_{lm}\left(\hat{r-r_{ni}}\right),\\ Y_{lm}\left(\hat{r-r_{ni}}\right)=Y_{lm}\left(\theta ,\varphi \right), \end{array}$$

where θ,φ are the angular spherical coordinates of the vector $r-r_{ni}.$

Here, $\delta =\tilde{\varepsilon }lm$ is an index that incorporates the quantum numbers for the energy value $\tilde{\varepsilon }$, the angular momentum quantum numbers are *l* and *m*, **r** is the electron position vector, and $r_{ni}$ is the position vector for the atom at site (*ni*) in equilibrium

(13) $$\begin{array}{l} r_{ni}=r_{n}+\rho_{i},\\ r_{n}=\sum_{\nu }l_{\nu }a_{\nu }, \end{array}$$

$r_{n}$ is the position vector of the unit cell n of the crystal lattice, and $\rho_{i}$ is the vector of the relative position of the node of the sublattice *i* in the unit cell n. The coordinates $l_{\nu }$ of the radius vector $r_{n}$ of the unit cell n of the crystal lattice are integers. The number ν takes on values ν=1,2,3 for three-dimensional crystals, ν=1,2 for two-dimensional crystals, and ν=1 for one-dimensional crystals.

Basis orthogonalization is performed with the Lowdin method [28]

(14) $$|{\tilde{\psi }}_{ni\delta }\rangle =S^{-1/2}|\psi_{ni\delta }\rangle ,S_{ni\delta ,n^{\prime }i^{\prime }\delta^{\prime }}=\langle \psi_{ni\delta }|\psi_{n^{\prime }i^{\prime }\delta^{\prime }}\rangle ,$$

where $S_{ni\delta ,n^{\prime }i^{\prime }\delta^{\prime }}$ are the overlapping matrix.

Vane’s functions $\phi_{ni\gamma }\left(r,\sigma^{\prime }\right)$, on which the Hamiltonian of the system are represented as in Equation (1), are defined from equation:

(15) $$\phi_{ni\gamma }\left(r,\sigma^{\prime }\right)={\tilde{\psi }}_{ni\delta }\left(r-r_{ni}\right)\chi_{\sigma }\left(\sigma^{\prime }\right)$$

where $\chi_{\sigma }(\sigma^{\prime })$ is the spin part of wave function, γ=δσ.

The orthogonalized wave function can be represented as:

(16) ψ˜n1i1δ1(r1,θ1,φ1)=∑n2,i2δ2Sn2i2δ2,n1i1δ1−12Rε˜2l2(r2)Yl2m2(θ2,φ2).

In expression (16):

(17) r1=r−rn1i1,r2=r−rn2i2=r1−rn2i2n1i1,r2=((x1−xn2i2n1i11)2+(x2−xn2i2n1i12)2+(x3−xn2i2n1i13)2)12,x1=r1sinθ1cosφ1,x2=r1sinθ1sinφ1,x3=r1cosθ1,xn2i2n1i1α=∑ν(lν(2)−lν(1))aνα+ρi2α−ρi1α,

(18) $$\cos\theta_{2}=\frac{r_{1}\cos\theta_{1}-x_{n_{2}i_{2}n_{1}i_{1}}^{3}}{r_{2}},$$

(19) φ2=arccosr1sinθ1cosφ1−xn2i2n1i11r2(1−cos2θ2)12.

Summation over $n_{2}i_{2}$ in expression (16) means summation over $r_{n_{2}i_{2}}$, in accordance with Formula (13).

The overlap matrix $S_{ni\delta ,n^{\prime }i^{\prime }\delta^{\prime }}$ is found from the equation:

(20) $$\begin{array}{l} S_{n_{1}i_{1}\delta_{1},n_{2}i_{2}\delta_{2}}=\\ ∭R_{{\tilde{\varepsilon }}_{1}l_{1}}\left(r_{1}\right)Y_{l_{1}m_{1}}^{*}\left(\theta_{1},\varphi_{1}\right)R_{{\tilde{\varepsilon }}_{2}l_{2}}\left(r_{2}\right)Y_{l_{2}m_{2}}\left(\theta_{2},\varphi_{2}\right)r_{1}^{2}\sin\theta_{1}dr_{1}d\theta_{1}d\varphi_{1} \end{array}$$

where $r_{2},\theta_{2},\varphi_{2}$ are expressed through $r_{1},\theta_{1},\varphi_{1}$ in accordance with Formulas (17)–(19).

To find matrix Sn2i2δ2,n1i1δ1−12 in expression (16), we find the Fourier transform of the matrix (20):

(21) $$S_{i_{1}\delta_{1},i_{2}\delta_{2}}\left(k\right)=\sum_{n_{2}}S_{n_{1}i_{1}\delta_{1},n_{2}i_{2}\delta_{2}}e^{ik(r_{n_{2}i_{2}}-r_{n_{1}i_{1}})}.$$

The vector k is defined by the expression

(22) $$\begin{array}{l} k=\sum_{\nu }k_{\nu }b_{\nu },\\ \left(a_{\nu }b_{\nu^{\prime }}\right)=2\pi \delta_{\nu \nu^{\prime }}, \end{array}$$

$b_{\nu }$ is the basis vector of the translations of the reciprocal lattice.

Summing over $n_{2}$ on the right-hand side of Formula (21) is easy to do if we replace it according to (13) and use

(23) $$k\left(r_{n_{2}i_{2}}-r_{n_{1}i_{1}}\right)=\sum_{\alpha =1}^{3}\sum_{\nu^{\prime }}k_{\nu^{\prime }}b_{\nu^{\prime }}^{\alpha }\left(\sum_{\nu }\left(l_{\nu }^{\left(2\right)}-l_{\nu }^{\left(1\right)}\right)a_{\nu }^{\alpha }+\rho_{i_{2}}^{\alpha }-\rho_{i_{1}}^{\alpha }\right).$$

As the matrix element $S_{n_{1}i_{1}\delta_{1},n_{2}i_{2}\delta_{2}}$ decreases with the distance between the nodes $n_{1}i_{1}$, $n_{2}i_{2}$, in numerical calculations, when summing over $n_{2}$ in expression (21), it is sufficient to restrict ourselves to a few coordination spheres. In this case, summation over $n_{2}$ is reduced to summation over $l_{\nu }^{\left(2\right)}$.

The matrix $S_{n_{1}i_{1}\delta_{1},n_{2}i_{2}\delta_{2}}$ has an infinite rank. The rank of the matrix $S_{i_{1}\delta_{1},i_{2}\delta_{2}}\left(k\right)$ is finite, which allows for finding the matrix Si1δ1,i2δ2−12(k). The matrix Sn2i2δ2,n1i1δ1−12 in expression (16) is found from equation:

(24) Sn1i1δ1,n2i2δ2−12=1N∑kSi1δ1,i2δ2−12(k)e−ik(rn2i2−rn1i1).

The values $h_{n_{1}i_{1}\gamma_{1},n_{2}i_{2}\gamma_{2}}^{\left(0\right)}$ in Equation (3) are the matrix elements of the kinetic and potential energy $\sum_{ni}v^{\lambda i}\left(r-r_{ni}\right)$ of the electron in the field of the cores of the atom’s ideal ordered crystal. The values $h_{n_{1}i_{1}\gamma_{1},n_{2}i_{2}\gamma_{2}}^{\left(0\right)}$ are defined by the expression:

(25) hn1i1γ1,n2i2γ2(0)=∑n3i3δ3Ei1ε˜1Sn3i3δ3,n1i1δ1−12∗Sn3i3δ3,n2i2δ212δσ1,σ2+∑n3i3≠n1i1vn1i1γ1,n2i2γ2n3i3,γ=δσ.

In the Formula (25)

(26) $$\begin{array}{l} v_{n_{1}i_{1}\gamma_{1},n_{2}i_{2}\gamma_{2}}^{n_{3}i_{3}}=\\ ∭{\tilde{\psi }}_{n_{1}i_{1}\delta_{1}}^{*}\left(r_{1},\theta_{1},\varphi_{1}\right)v^{i_{3}}\left(r_{3}\right){\tilde{\psi }}_{n_{2}i_{2}\delta_{2}}\left(r_{2},\theta_{2},\varphi_{2}\right)r_{1}^{2}\sin\theta_{1}dr_{1}d\theta_{1}d\varphi_{1}\times \delta_{\sigma_{1},\sigma_{2}} \end{array},\;E_{i_{1}{\tilde{\varepsilon }}_{1}}=-\frac{me^{4}{\left(Z_{i_{1}}\right)}^{2}}{2\hbar^{2}{\tilde{\varepsilon }}_{1}^{2}},{\tilde{\varepsilon }}_{1}=1,2,3,\ldots ,$$

Here, $r_{2},\theta_{2},\varphi_{2}$ is expressed through $r_{1},\theta_{1},\varphi_{1}$ in accordance with Formulas (17)–(19). The expression for $r_{3}$ is obtained from expression (17) for $r_{2}$ replacement $x_{n_{2}i_{2}n_{1}i_{1}}^{\alpha }$ by $x_{n_{3}i_{3}n_{1}i_{1}}^{\alpha }$. Summation over $n_{3}i_{3}$ in expression (25) means summation over $r_{n_{3}i_{3}}$, in accordance with Formula (13).

In Formula (26) and e are the mass and charge of the electron, respectively, and $Z_{i}$ is the ordinal number of an atom of the sort λ located in the site ni of an ideally ordered crystal. ℏ denotes the Planck’s constant.

The matrix element of the electron–ion interaction Hamiltonian in Equation (6) is given by

(27) $$w_{ni\gamma ,n^{\prime }i^{\prime }\gamma^{\prime }}=\sum_{n^{\prime \prime }i^{\prime \prime }}w_{ni\gamma ,n^{\prime }i^{\prime }\gamma^{\prime }}^{n^{\prime \prime }i^{\prime \prime }}$$

where

(28) $$w_{ni\gamma ,n^{\prime }i^{\prime }\gamma^{\prime }}^{n^{\prime \prime }i^{\prime \prime }}=\sum_{\lambda }c_{n^{\prime \prime }i^{\prime \prime }}^{\lambda }\;w_{ni\gamma ,n^{\prime }i^{\prime }\gamma^{\prime }}^{\lambda n^{\prime \prime }i^{\prime \prime }},$$

(29) $$w_{ni\gamma ,n^{\prime }i^{\prime }\gamma^{\prime }}^{\lambda n^{\prime \prime }i^{\prime \prime }}=v_{ni\gamma ,n^{\prime }i^{\prime }\gamma^{\prime }}^{\lambda n^{\prime \prime }i^{\prime \prime }}+\Delta v_{ni\gamma ,n^{\prime }i^{\prime }\gamma^{\prime }}^{\lambda n^{\prime \prime }i^{\prime \prime }}-v_{ni\gamma ,n^{\prime }i^{\prime }\gamma^{\prime }}^{i^{\prime \prime }}$$

$v_{n_{1}i_{1}\gamma_{1},n_{2}i_{2}\gamma_{2}}^{\lambda n^{\prime \prime }i^{\prime \prime }}$ is a matrix element of the potential of the core of the atom $v^{\lambda }\left(r-r_{n^{\prime \prime }i^{\prime \prime }}\right)$.

The expression for $v_{n_{1}i_{1}\gamma_{1},n_{2}i_{2}\gamma_{2}}^{\lambda n_{3}i_{3}}$ is obtained from Formula (26) by replacing $v^{i_{3}}\left(r_{3}\right)$ with $v^{\lambda }\left(r_{3}\right)$.

In Equation (28), $c_{ni}^{\lambda }$ is a discrete binary random number taking the values of 1 or 0, depending on whether an atom of type λ is at site (*ni*) or not, respectively. The symbol $\Delta v_{ni\gamma ,n^{\prime }i^{\prime }\gamma^{\prime }}^{\lambda n^{\prime \prime }i^{\prime \prime }}$ will be defined next.

The expression for the electron–phonon interaction in Equation (7) is found through derivatives of the potential energy of the electrons in the ion core field due to a displacement of the atom by vector $u_{ni}$. In Equation (7), the value of $v_{\;ni\gamma ,n^{\prime }i^{\prime }\gamma^{\prime }}^{\prime }$ is given by

(30) $$v_{\;ni\gamma ,n^{\prime }i^{\prime }\gamma^{\prime }}^{\prime }=\sum_{n^{\prime \prime }i^{\prime \prime }\alpha }v^{\prime }{}_{ni\gamma ,n^{\prime }i^{\prime }\gamma^{\prime }}^{n^{\prime \prime }i^{\prime \prime }\alpha }u_{n^{\prime \prime }i^{\prime \prime }\alpha },$$

where

(31) $$v^{\prime }{}_{ni\gamma ,n^{\prime }i^{\prime }\gamma^{\prime }}^{n^{\prime \prime }i^{\prime \prime }\alpha }=\sum_{\lambda }c_{n^{\prime \prime }i^{\prime \prime }}^{\lambda }\;v^{\prime }{}_{ni\gamma ,n^{\prime }i^{\prime }\gamma^{\prime }}^{\lambda n^{\prime \prime }i^{\prime \prime }\alpha }$$

with $v^{\prime }{}_{ni\gamma ,n^{\prime }i^{\prime }\gamma^{\prime }}^{\lambda n^{\prime \prime }i^{\prime \prime }\alpha }$ the matrix elements of the following operator:

(32) $$-e_{n^{\prime \prime }i^{\prime \prime }\alpha }\frac{d}{d\left|r-r_{n^{\prime \prime }i^{\prime \prime }}\right|}v^{\lambda i^{\prime \prime }}\left(\left|r-r_{n^{\prime \prime }i^{\prime \prime }}\right|\right),\;e_{n^{\prime \prime }i^{\prime \prime }}=\frac{r-r_{n^{\prime \prime }i^{\prime \prime }}}{\left|r-r_{n^{\prime \prime }i^{\prime \prime }}\right|}.$$

The expression for $v^{\prime }{}_{n_{1}i_{1}\gamma_{1},n_{2}i_{2}\gamma_{2}}^{\lambda n_{3}i_{3}\alpha }$ is obtained from Formula (26) by replacing in it $v^{i_{3}}\left(r_{3}\right)$ with

(33) $$-\frac{\left(x_{\alpha }-x_{n_{3}i_{3}n_{1}i_{1}}^{\alpha }\right)}{r_{3}}\frac{d}{dr_{3}}v^{\lambda n_{3}i_{3}}\left(r_{3}\right).$$

$\Delta v_{ni\gamma ,n^{\prime }i^{\prime }\gamma^{\prime }}^{\lambda n^{\prime \prime }i^{\prime \prime }}$ in Equation (29) describes electron scattering on the static displacement of the atoms, and is defined by the equation

(34) $$\Delta v_{ni\gamma ,n^{\prime }i^{\prime }\gamma^{\prime }}^{\lambda n^{\prime \prime }i^{\prime \prime }}=\sum_{\alpha }v^{\prime }{}_{ni\gamma ,n^{\prime }i^{\prime }\gamma^{\prime }}^{\lambda n^{\prime \prime }i^{\prime \prime }\alpha }u_{n^{\prime \prime }i^{\prime \prime }\alpha }^{s,\lambda }$$

where $u_{n^{\prime \prime }i^{\prime \prime }\alpha }^{s,\lambda }$ is the α projection of the static displacement of the atom of type λ in the site, and $n^{\prime \prime }i^{\prime \prime }$ I caused by the difference in the atomic radii of the components of the disordered crystal.

Upon receipt of expressions (27)–(34), it was taken into account that the potential energy operator of the electron in the field of the atoms core can be expressed as:$v^{ni}\left(r-{r^{\prime }}_{ni}\right)$, $r\prime_{ni}=r_{ni}+u_{ni}^{s}+u_{ni}$, with **r** being the electron’s radius vector, $r_{ni}$ the radius-vector of atom’s equilibrium position in the site of the crystal lattice (*ni*), $u_{ni}^{s}$ the vector of atom’s static displacement from equilibrium position in site (*ni*), and $u_{ni}$ the atom’s displacement operator in site (*ni)*. Expanding $v^{ni}\left(r-{r^{\prime }}_{ni}\right)$ in the series in powers $u_{ni\alpha }$ and restricting ourselves to linear terms, we arrive at expressions (27)–(34).

The matrix of the force constants arising from the direct Coulomb interaction of the ionic cores has the form:

(35) $$\begin{array}{l} \Phi_{ni\alpha ,n^{\prime }i^{\prime }\alpha^{\prime }}=-\frac{Z_{ni}Z_{n^{\prime }i^{\prime }}e^{2}}{4\pi \varepsilon_{0}{\left|r_{n}+\rho_{i}-r_{n^{\prime }}-\rho_{i^{\prime }}\right|}^{5}}\times \\ \times [3\left(r_{n\alpha }+\rho_{i\alpha }-r_{n^{\prime }\alpha }-\rho_{i^{\prime }\alpha }\right)\left(r_{n\alpha^{\prime }}+\rho_{i\alpha^{\prime }}-r_{n^{\prime }\alpha^{\prime }}-\rho_{i^{\prime }\alpha^{\prime }}\right)-\\ -{\left|r_{n}+\rho_{i}-r_{n^{\prime }}-\rho_{i^{\prime }}\right|}^{2}\delta_{\alpha \alpha^{\prime }}],ni\neq n^{\prime }i^{\prime }. \end{array}$$

where $Z_{ni}$ is the serial number of the atom located in the lattice site ni of the disordered crystal, which is given by the expression

(36) $$Z_{ni}=\sum_{\lambda }c_{ni}^{\lambda }\;Z_{i}.$$

This matrix $\Phi_{ni\alpha ,n^{\prime }i^{\prime }\alpha^{\prime }}$ satisfies the following constraint:

(37) $$\sum_{n^{\prime }i^{\prime }}\Phi_{ni\alpha ,n^{\prime }i^{\prime }\alpha^{\prime }}=0.$$

Multicenter integrals $v_{n_{3},n_{4}}^{\left(2\right)n_{1},n_{2}},n=\left(ni\gamma \right)$ in Formula (8) can be represented as

(38) $$\begin{array}{l} v_{n_{3}i_{3}\gamma_{3},n_{4}i_{4}\gamma_{4}}^{n_{1}i_{1}\gamma_{1},n_{2}i_{2}\gamma_{2}}=e^{2}\delta_{\sigma_{1}\sigma_{4}}\delta_{\sigma_{2}\sigma_{3}}\iint \frac{1}{\left|r^{\prime }-r^{\prime \prime }\right|}\\ \times {\tilde{\psi }}_{n_{1}i_{1}\delta_{1}}^{*}\left(r_{1}^{\prime },\theta_{1}^{\prime },\varphi_{1}^{\prime }\right){\tilde{\psi }}_{n_{2}i_{2}\delta_{2}}^{*}\left(r_{1}^{\prime \prime },\theta_{1}^{\prime \prime },\varphi_{1}^{\prime \prime }\right){\tilde{\psi }}_{n_{3}i_{3}\delta_{3}}\left(r_{2}^{\prime \prime },\theta_{2}^{\prime \prime },\varphi_{2}^{\prime \prime }\right){\tilde{\psi }}_{n_{4}i_{4}\delta_{4}}\left(r_{2}^{\prime },\theta_{2}^{\prime },\varphi_{2}^{\prime }\right)\\ \times d^{3}r_{1}^{\prime }d^{3}r_{1}^{\prime \prime }. \end{array}$$

In Formula (38)

(39) |r′−r″|=(∑α(x′α−x″α−xn2i2n1i1α)2)12,

(40) $$d^{3}r_{1}^{\prime }=r_{1}^{\prime }{}^{2}\sin\theta_{1}^{\prime }dr_{1}^{\prime }d\theta_{1}^{\prime }d\varphi_{1}^{\prime },$$

When integrating over $r_{1}^{\prime },\theta_{1}^{\prime },\varphi_{1}^{\prime }$ in Formula (38), $r_{2}^{\prime },\theta_{2}^{\prime },\varphi_{2}^{\prime }$ should be expressed through $r_{1}^{\prime },\theta_{1}^{\prime },\varphi_{1}^{\prime }$, in accordance with Formulas (17)–(19), in which I is necessary to replace $x_{n_{2}i_{2}n_{1}i_{1}}^{\alpha }$ with $x_{n_{4}i_{4}n_{1}i_{1}}^{\alpha }$. When integrating over $r_{1}^{\prime \prime },\theta_{1}^{\prime \prime },\varphi_{1}^{\prime \prime }$ in Formula (38), $r_{2}^{\prime \prime },\theta_{2}^{\prime \prime },\varphi_{2}^{\prime \prime }$ should be expressed through $r_{1}^{\prime \prime },\theta_{1}^{\prime \prime },\varphi_{1}^{\prime \prime }$, in accordance with Formulas (17)–(19), in which it is necessary to replace $x_{n_{2}i_{2}n_{1}i_{1}}^{\alpha }$ with $x_{n_{3}i_{3}n_{2}i_{2}}^{\alpha }$.

So, Formulas (17)–(19) describe the procedure for calculating the matrix elements $h_{n_{1}i_{1}\gamma_{1},n_{2}i_{2}\gamma_{2}}^{\left(0\right)}$, $v_{n_{3}i_{3}\gamma_{3},n_{4}i_{4}\gamma_{4}}^{\left(2\right)n_{1}i_{1}\gamma_{1},n_{2}i_{2}\gamma_{2}}$ Hamiltonian (1), containing one-electron and two-electron integrals.

## 3. Green’s Functions of Electrons and Phonons

We employ a Green’s function-based formalism to perform the calculations. Ultimately, we need the real-time retarded $G_{r}^{AB}(t,t^{\prime })$ and advanced $G_{a}^{AB}(t,t^{\prime })$ Green’s functions are each defined as follows [26]:

(41) $$G_{r}^{AB}(t,t^{\prime })=-\frac{i}{\hbar }\theta (t-t^{\prime })<[\tilde{A}(t),\tilde{B}(t^{\prime })]>,\;G_{a}^{AB}(t,t^{\prime })=\frac{i}{\hbar }\theta (t^{\prime }-t)<[\tilde{A}(t),\tilde{B}(t^{\prime })]>.$$

Here, the operators are expressed in the Heisenberg representation

(42) $$\tilde{A}(t)=e^{iH\;t/\hbar }A\;e^{-iH\;t/\hbar },$$

where ℏ is Planck’s constant, $H=H-\mu_{e}N_{e}$, $\mu_{e}$ is the chemical potential of the electronic subsystem, and $N_{e}$ is the electron number operator given by

(43) $$N_{e}=\sum_{ni\gamma }a_{ni\gamma }^{+}a_{ni\gamma }^{}.$$

In addition, the commutator or anticommutator is defined via

(44) [A,B]=AB∓ BA,

where the commutator is used for Bose operators (−) and the anticommutator is used for Fermi operators (+). The symbol θ(t) is Heaviside’s unit step function. The angle brackets 〈…〉 denote the thermal averaging with respect to the density matrix ρ

(45) $$<A>=Tr\;(\rho A),\;\rho =e^{(\Omega -H)/\Theta },$$

where Ω is the thermodynamic potential of the system given by exp(Ω/Θ) = Trexp(−H/Θ) and $\Theta =k_{b}T$, with *k_b_* Boltzmann’s constant and T the temperature.

The thermal Green’s functions are defined by

(46) $$G_{}^{AB}(\tau ,\tau^{\prime })=-<T_{\tau }\tilde{A}(\tau )\tilde{B}(\tau^{\prime })>,$$

where the imaginary-time operator $\tilde{A}(\tau )$ is derived from the real-time Heisenberg representation and the substitution t=−iℏτ. Hence,

(47) $$\tilde{A}(\tau )=e^{H\;\tau }A\;e^{-H\;\tau }.$$

In addition, the time-ordering operator satisfies

(48) $$\begin{array}{l} T_{\tau }\tilde{A}(\tau )\tilde{B}(\tau^{\prime })=\theta (\tau -\tau^{\prime })\tilde{A}(\tau )\tilde{B}(\tau^{\prime })+\\ \pm \theta (\tau^{\prime }-\tau )\tilde{B}(\tau^{\prime })\tilde{A}(\tau ) \end{array},$$

where the plus sign is used for Bose operators and the minus sign for Fermi operators.

We next go to the interaction representation by introducing the operator

(49) $$\sigma (\tau )=e^{H_{0}\;\tau }e^{-H\;\tau },$$

with $H=H_{0}+H_{\mathrm{int}}$ and $H_{0}=H_{0}-\mu_{e}N_{e}$.

Differentiating the expression for σ(τ) in Equation (68) with respect to τ and then integrating starting from 0, with the boundary condition σ(0)=1, we obtain:

(50) $$\sigma (\tau )=T_{\tau }\exp\left[-\int_{0}^{\tau }H_{\mathrm{int}}(\tau^{\prime })d\tau^{\prime }\right],$$

where $H_{\mathrm{int}}(\tau )=e^{H_{0}\;\tau }H_{\mathrm{int}}\;e^{-H_{0}\;\tau }$. Employing this result yields

(51) $$\tilde{A}(\tau )=\sigma^{-1}(\tau )A(\tau )\sigma (\tau ),$$

with *A*(τ) in the Heisenberg representation with respect to the noninteracting Hamiltonian. Substituting these results into the definition of the thermal Green’s function creates the alternate interaction-representation form for the Green’s function, given by

(52) $$G^{AB}(\tau ,\tau^{\prime })=-\frac{<T_{\tau }A(\tau )B(\tau^{\prime })\sigma (1/\Theta ){>}_{0}}{<\sigma (1/\Theta ){>}_{0}}$$

where all time dependence is with respect to the noninteracting Hamiltonian and the trace over all states is with respect to the noninteracting states

(53) $$<A{>}_{0}=Tr(\rho_{0}A),\;\rho_{0}=e^{(\Omega_{0}^{}-H_{0})/\Theta }.$$

Next, we expand expression (51) for σ(1/θ) in a series in powers of the interaction Hamiltonian $H_{\mathrm{int}}^{}(\tau )$ and substitute this expression in Formula (53).

The diagrammatic method is generated by expanding σ(τ) in a power series in terms of $H_{\mathrm{int}}^{}(\tau )$, and then using Wick’s theorem to evaluate the resulting operator averages.

The numbers of quantum states for different operators in the interaction Hamiltonian $H_{\mathrm{int}}^{}(\tau )$ (5)–(11), (51) are different, and the values of the argument τ are the same.

Each operator can be assigned a quantum state number and an argument number τ, if in expression (51) for σ(1/θ) the operator $H_{\mathrm{int}}(\tau )$ is replaced by an operator $H_{\mathrm{int}}(\tau ,\tau_{1},\ldots ,\tau_{k})$ in which the values of the argument τ for operators with different quantum states are different, the matrix elements differ from the matrix elements of the operator $H_{\mathrm{int}}(\tau )$ by a factor $\delta (\tau -\tau_{1})\ldots \delta (\tau -\tau_{k})$, and the single integral over τ is replaced by the integral over $\tau ,\tau_{1},\ldots ,\tau_{k}$ multiplicity k+1. The multiplicity of the integral is different for different types of interaction. In expression (53) for $G^{AB}(\tau ,\tau^{\prime })$, the term of the series for σ(1/θ) (51) forms a multiple sum over quantum states and an integral over τ of the mean T-product of operators $H_{\mathrm{int}}(\tau ,\tau_{1},\ldots ,\tau_{k})$ multiplied by an operator $A\left(\tau \right)B\left(\tau^{\prime }\right)$. The T-product of operators is averaged over the occupation numbers of the quantum states of the system of noninteracting electrons and phonons, in accordance with Formula (53). The numbers of the quantum states for the operators in the indicated T-product are ordered by the matrix elements of the interaction operators $H_{\mathrm{int}}(\tau ,\tau_{1},\ldots ,\tau_{k})$, in accordance with Formulas (5)–(11), in such a way that pairs of operators are formed. This is due to the fact that among the average T-products of operators, only those in which the number of operators is even for the electron subsystem and the phonon subsystem are nonzero. The quantum state for each operator of the pair, except for the operators A(τ), $B\left(\tau^{\prime }\right)$, coincides with one of the quantum states for the corresponding matrix element of the interaction operators $H_{\mathrm{int}}(\tau ,\tau_{1},\ldots ,\tau_{k})$ in the given product.

Let us give the averaging technique in expression (71) a simpler form. For this, in the T-product of each pair of operators $a_{n_{1}}\left(\tau_{1}\right)a_{n_{2}}^{+}\left(\tau_{2}\right),n=\left(ni\gamma \right)$ for the electron subsystem and $u_{n_{1}}\left(\tau_{1}\right)u_{n_{2}}\left(\tau_{2}\right)$, $P_{n_{1}}\left(\tau_{1}\right)P_{n_{2}}\left(\tau_{2}\right)$, $u_{n_{1}}\left(\tau_{1}\right)P_{n_{2}}\left(\tau_{2}\right)$, $P_{n_{1}}\left(\tau_{1}\right)u_{n_{2}}\left(\tau_{2}\right)$, n=(niα) for the phonon subsystem, in the Hamiltonian of the system of noninteracting electrons and phonons $H_{0}$ (2)–(4), (53), we compare the sum of the products of pairs of operators $H_{0n_{1}n_{2}}$, the numbers of quantum states of which coincide with the numbers of quantum states depending on τ the operators of the pair.

Provided that the numbers of quantum states for the operators in the T-product are ordered by the matrix elements of the interaction operators $H_{\mathrm{int}}(\tau ,\tau_{1},\ldots ,\tau_{k})$ standing in it, the operators $\exp\left(-H_{0n_{1}n_{2}}/\theta \right)$, $\exp\left(-H_{0n_{1}n_{2}}\tau \right)$ change places with the products depending on τ other pairs of operators. It follows from this that the average of the T-product of several operators in expression (53) is equal to the product of the average T-products of pairs of operators that determine the matrices of the Green’s functions for the zero-order Hamiltonian $H_{0}$. This statement also extends to the case when the quantum states for the operators of a pair coincide with the quantum states for the operators of other pairs. This follows from the fact that the distribution function of a system of an infinite number of particles over the occupation numbers of quantum states has a sharp maximum, and the most probable value of a physical quantity is equal to its average value. The quantum state $n_{i}$ and the argument $\tau_{i}$ for each operator of the pair, except for the operators A(τ), $B\left(\tau^{\prime }\right)$, coincides with one of the quantum states $n_{i}$ and arguments $\tau_{i}$ for the corresponding matrix element of the interaction operators $H_{\mathrm{int}}(\tau ,\tau_{1},\ldots ,\tau_{k})$ in the given product. In expression (71), the Green’s function $G^{AB}(\tau ,\tau^{\prime })$ is summed over quantum states $n_{i}$ and integrated over arguments $\tau_{i}$.

The averaging technique described above in expression (71) for the Green’s function $G^{AB}(\tau ,\tau^{\prime })$ is the essence of Wick’s theorem. This technique then generalizes the approach used for the homogeneous system [27].

The technique for calculating the Green’s function $G^{AB}(\tau ,\tau^{\prime })$ (53) becomes clearer if the terms on the right-hand side of Equation (53) are represented in the form of diagrams. If the Green’s function of the system is expressed as a series only over connected diagrams [27], then the denominator in Formula (53) will cancel out with the same factor in the numerator. So, the thermal Green’s function is expanded in terms of connected diagrams. The indicated diagrammatic series can be easily summed up, which makes it possible to go beyond the framework of the first approximations of the perturbation theory and obtain equations for the Green’s functions of the system.

Summing up the indicated series, using the standard relation between the spectral representations of the temperature and real-time Green’s functions and performing an analytical continuation on the real axis, we obtain the following equations for the retarded Green’s functions [26] (hereinafter the dependence on r is suppressed):

(54) $$G^{aa^{+}}(\varepsilon )=G_{0}^{aa^{+}}(\varepsilon )+G_{0}^{aa^{+}}(\varepsilon )\;\left(w+\Sigma_{eph}(\varepsilon )+\Sigma_{ee}(\varepsilon )\right)\;G_{}^{aa^{+}}(\varepsilon )\;\begin{array}{l} G^{uu}(\varepsilon )=G_{0}^{uu}(\varepsilon )+G_{0}^{uu}(\varepsilon )\;\left(\Delta \Phi +\Sigma_{phe}(\varepsilon )+\Sigma_{phph}(\varepsilon )\right)\times \\ \times G_{}^{uu}(\varepsilon )+G_{0}^{uP}(\varepsilon )\Delta M^{-1}G_{}^{Pu}(\varepsilon ), \end{array}\;\begin{array}{l} G^{PP}(\varepsilon )=G_{0}^{PP}(\varepsilon )+G_{0}^{PP}(\varepsilon )\Delta M^{-1}G_{}^{PP}(\varepsilon )+G_{0}^{Pu}(\varepsilon )\times \\ \times \left(\Delta \Phi +\Sigma_{phe}(\varepsilon )+\Sigma_{phph}(\varepsilon )\right)\;G_{}^{uP}(\varepsilon ), \end{array}\;\begin{array}{l} G^{uP}(\varepsilon )=G_{0}^{uP}(\varepsilon )+G_{0}^{uP}(\varepsilon )\Delta M^{-1}G_{}^{PP}(\varepsilon )+G_{0}^{uu}(\varepsilon )\times \\ \times \left(\Delta \Phi +\Sigma_{phe}(\varepsilon )+\Sigma_{phph}(\varepsilon )\right)\;G_{}^{uP}(\varepsilon ), \end{array}\;\begin{array}{l} G^{Pu}(\varepsilon )=G_{0}^{Pu}(\varepsilon )+G_{0}^{Pu}(\varepsilon )\;\left(\Delta \Phi +\Sigma_{phe}(\varepsilon )+\Sigma_{phph}(\varepsilon )\right)\times \\ \times G_{}^{uu}(\varepsilon )+G_{0}^{PP}(\varepsilon )\Delta M^{-1}G_{}^{Pu}(\varepsilon ), \end{array}$$

where ε=ℏω. Here, $G^{aa^{+}}(\varepsilon )$, $G^{uu}(\varepsilon )$, $G^{PP}(\varepsilon )$, $G^{uP}(\varepsilon )$, $G^{Pu}(\varepsilon )$ are the real-frequency representation of the single-particle Green’s function of the electrons, the coordinate-coordinate, momentum–momentum, coordinate–momentum, and momentum–coordinate Green’s functions of the phonons, respectively; and $\Sigma_{eph}(\varepsilon )$, $\Sigma_{phe}(\varepsilon )$, $\Sigma_{ee}(\varepsilon )$, $\Sigma_{phph}(\varepsilon )$ are the corresponding self-energies (mass operators) for the electron–phonon, phonon–electron, electron–electron, and phonon–phonon interactions.

The real-time and real-frequency Green’s functions are related by standard Fourier transform relations given by

(55) $$G_{r,a}^{AB}(t)=\frac{1}{2\pi }\int_{-\infty }^{\infty }G_{r,a}^{AB}(\omega )\;e^{-i\omega t}d\omega$$

and

(56) $$G_{r,a}^{AB}(\omega )=\int_{-\infty }^{\infty }G_{r,a}^{AB}(t)\;e^{i\omega t}dt.$$

The thermal Green’s functions are periodic (bosons) or antiperiodic (fermions) on the interval −1/Θ ≤ τ < 1/Θ, and hence have a Fourier series representation in terms of their Matsubara frequencies, as follows:

(57) $$G^{AB}(\tau )=\Theta \sum_{\omega_{n}}G^{AB}(\omega_{n})\;e^{-i\omega_{n}\tau }$$

and

(58) $$G^{AB}(\omega_{n})=\frac{1}{2}\int_{-1/\Theta }^{1/\Theta }G^{AB}(\tau )\;e^{i\omega_{n}\tau }d\tau ,$$

where the Matsubara frequencies satisfy

(59) $$\begin{array}{l} \omega_{n}=\{\begin{array}{cc} 2n\pi \;\Theta & \mathrm{for}\;\mathrm{Bose}\;\mathrm{particles},\\ (2n+1)\pi \;\Theta & \mathrm{for}\;\mathrm{Fermi}\;\mathrm{particles}, \end{array}\;\\ n=0,\;\pm 1,\;\pm 2,\ldots \end{array}$$

The electronic Green’s functions are infinite matrices with indices given by the lattice site *n*, the basis site *i*, and the other quantum numbers γ. Similarly, the phonon Green’s functions also are infinite matrices with the same lattice and basis site dependence, plus a dependence on the spatial coordinate direction α. Using the equations of motion for Green’s functions, one can obtain simple expressions for the zero-order Green’s functions, namely [26]:

(60) $$G_{0}^{aa^{+}}(\varepsilon )={\left[\varepsilon -H_{0}^{\left(1\right)}\right]}^{-1},$$

with

(61) $$H_{0}^{\left(1\right)}=\left\|h_{ni\gamma ,n^{\prime }i^{\prime }\gamma^{\prime }}^{\left(0\right)}\right\|,$$

(62) $$G_{0}^{uu}(\varepsilon )={\left[\omega^{2}M^{\left(0\right)}-\Phi^{\left(0\right)}\right]}^{-1},$$

with

(63) $$\Phi^{\left(0\right)}=\left\|\Phi^{\left(0\right)}{}_{ni\alpha ,n^{\prime }i^{\prime }\alpha^{\prime }}\right\|$$

and

(64) $$M^{\left(0\right)}=\left\|M_{i}\delta_{nn^{\prime }}\delta_{ii^{\prime }}\delta_{\alpha \alpha^{\prime }}\right\|.$$

Here, the double lines denote a matrix.

When the perturbations are small, given by

(65) $$\frac{{\left(\frac{\varepsilon^{2}}{\hbar^{2}}\Delta M+\Delta \Phi +\Sigma_{phe}(\varepsilon )+\Sigma_{phph}(\varepsilon )\right)}_{ni\alpha ,n^{\prime }i^{\prime }\alpha^{\prime }}}{\Phi_{ni\alpha ,n^{\prime }i^{\prime }\alpha^{\prime }}^{\left(0\right)}}<<1,$$

then the solution of the system of equations in Equation (55) becomes

(66) $$G^{aa^{+}}(\varepsilon )={\left[\varepsilon -H_{0}^{\left(1\right)}-\left(w+\Sigma_{eph}(\varepsilon )+\Sigma_{ee}(\varepsilon )\right)\right]}^{-1},$$

(67) $$G^{uu}(\varepsilon )={\left[\omega^{2}M^{\left(0\right)}-\Phi^{\left(0\right)}-\left(\frac{\varepsilon^{2}}{\hbar^{2}}\Delta M+\Delta \Phi +\Sigma_{phe}(\varepsilon )+\Sigma_{phph}(\varepsilon )\right)\right]}^{-1},$$

(68) $$G_{}^{PP}(\varepsilon )=\frac{\varepsilon^{2}}{\hbar^{2}}{\left(M^{\left(0\right)}\right)}^{2}\;G_{}^{uu}(\varepsilon ),$$

where

(69) $$\Delta M=\left\|(M_{i}-M_{ni})\delta_{nn^{\prime }}\delta_{ii^{\prime }}\delta_{\alpha \alpha^{\prime }}\right\|,\varepsilon =\hbar \omega .$$

**Figure 1:**
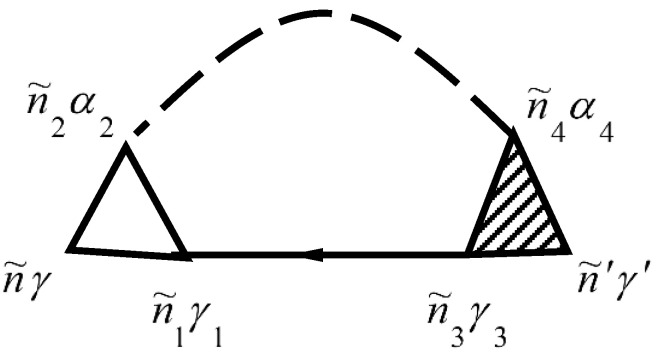
Diagram for $\Sigma_{eph\;ni\gamma ,n^{\prime }i^{\prime }\gamma^{\prime }}(\tau ,\tau^{\prime })=\Sigma_{eph\;\tilde{n}\gamma ,{\tilde{n}}^{\prime }\gamma^{\prime }}$. Here $\tilde{n}=(ni\tau )$.

The mass operator of the Green’s function of electrons for the electron–phonon interaction $\Sigma_{eph}(\tau ,\tau^{\prime })$ is described by the diagram in Figure 1. The mass operator of the Green’s function of electrons for the electron–phonon interaction $\Sigma_{eph}(\tau ,\tau^{\prime })$ is described by the diagram in [26,29,30].

**Figure 2:**
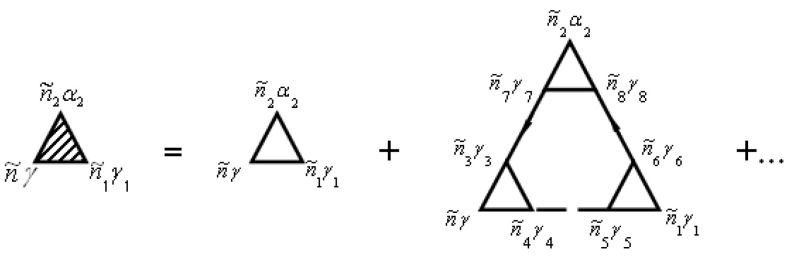
Diagrams for the vertex part $\Gamma_{ni\gamma ,n_{1}i_{1}\gamma_{1}}^{n_{2}i_{2}\alpha_{2}}(\tau_{2},\tau ,\tau_{1})=\Gamma_{\tilde{n}\gamma ,{\tilde{n}}_{1}\gamma_{1}}^{{\tilde{n}}_{2}\alpha_{2}}$. Here $\tilde{n}=(ni\tau )$.

Solid lines in Figure 1 correspond to the Green’s function of electrons $G_{ni\gamma ,n^{\prime }i^{\prime }\gamma^{\prime }}^{aa^{+}}(\tau ,\tau^{\prime })$ and dashed lines correspond to the Green’s function of phonons $G_{ni\alpha ,n^{\prime }i^{\prime }\alpha^{\prime }}^{uu}(\tau ,\tau^{\prime })$. The vertex part $\Gamma_{ni\gamma ,n_{1}i_{1}\gamma_{1}}^{n_{2}i_{2}\alpha_{2}}(\tau_{2},\tau ,\tau_{1})$ of the mass operator of the Green’s function is described by the diagrams in Figure 2.

The not shaded triangle in Figure 2 corresponds to equation

(70) $$\Gamma_{0\;ni\gamma ,n_{1}i_{1}\gamma_{1}}^{n_{2}i_{2}\alpha_{2}}(\tau_{2},\tau ,\tau_{1})=v^{\prime }{}_{ni\gamma ,n_{1}i_{1}\gamma_{1}}^{n_{2}i_{2}\alpha_{2}}\delta (\tau -\tau_{2})\;\delta (\tau -\tau_{1}).$$

In Figure 1 and Figure 2, summation for internal points $\tilde{n}\gamma$ is carried out. Summation of $\tilde{n}\gamma$ provides summation of niγ and integration over τ. Expressions that correspond to each diagram are attributed to multiplier ${\left(-1\right)}^{n+F}$, where n is the diagram’s order (namely the number of vertices $\Gamma_{0}$ in the diagram), and F is the number of lines for the Green’s function of electrons $G_{}^{aa^{+}}$. This function goes out and goes in in the same vertices.

Explicitly, the electron–phonon self-energy becomes

(71) $$\begin{array}{l} \Sigma_{eph\;ni\gamma ,n^{\prime }i^{\prime }\gamma^{\prime }}(\varepsilon )=-\frac{1}{4\pi i}\int_{-\infty }^{\infty }d\varepsilon^{\prime }\cot h\left(\frac{\varepsilon^{\prime }}{2\Theta }\right)\;\Gamma_{\;ni\gamma ,\;n_{3}i_{3}\gamma_{3}}^{\left(0\right)n_{1}i_{1}\alpha_{1}}\times \\ \times \left[G_{n_{1}i_{1}\alpha_{1},n_{2}i_{2}\alpha_{2}}^{uu}(\varepsilon^{\prime })-G_{n_{1}i_{1}\alpha_{1},n_{2}i_{2}\alpha_{2}}^{uu\;*}(\varepsilon^{\prime })\right]\;G_{n_{3}i_{3}\gamma_{3},n_{4}i_{4}\gamma_{4}}^{aa^{+}}\times \\ \times (\varepsilon -\varepsilon^{\prime })\Gamma_{\;n_{4}i_{4}\gamma_{4},n^{\prime }i^{\prime }\gamma^{\prime }}^{n_{2}i_{2}\alpha_{2}}, \end{array}$$

(72) $$\Gamma_{\;ni\gamma ,\;n_{3}i_{3}\gamma_{3}}^{\left(0\right)n_{1}i_{1}\alpha_{1}}=v^{\prime }{}_{ni\gamma ,\;n_{3}i_{3}\gamma_{3}}^{n_{1}i_{1}\alpha_{1}}.$$

where repeated indices are summed over.

**Figure 3:**
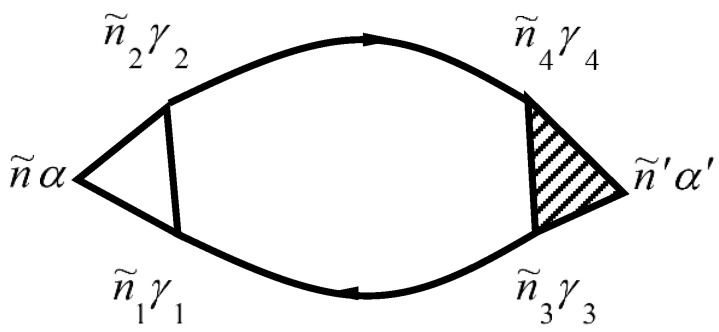
Diagram for $\Sigma_{phe\;ni\alpha ,n^{\prime }i^{\prime }\alpha^{\prime }}(\tau ,\tau^{\prime })=\Sigma_{phe\;\tilde{n}\alpha ,{\tilde{n}}^{\prime }\alpha^{\prime }}$. In Figure 3, $\tilde{n}=(ni\tau )$.

Phonon–electron interaction is described by the diagram in Figure 3. Phonon–electron interaction is described by the diagram in [29,30].

The designation in Figure 3 corresponds to designations in Figure 1 and Figure 2.

The self-energy of the phonon due to the phonon–electron interaction is given by

(73) $$\begin{array}{l} \Sigma_{phe\;ni\alpha ,n^{\prime }i^{\prime }\alpha^{\prime }}(\varepsilon )=\frac{1}{2\pi i}\int_{-\infty }^{\infty }d\varepsilon^{\prime }\;f(\varepsilon^{\prime })\Gamma_{n_{2}i_{2}\gamma_{2},n_{1}i_{1}\gamma_{1}}^{\;\left(0\right)ni\alpha }\times \\ \times \{\left[G_{n_{1}i_{1}\gamma_{1},n_{3}i_{3}\gamma_{3}}^{aa^{+}}(\varepsilon +\varepsilon^{\prime })-G_{n_{1}i_{1}\gamma_{1},n_{3}i_{3}\gamma_{3}}^{aa^{+}*}(\varepsilon +\varepsilon^{\prime })\right]\times \\ \times G_{n_{4}i_{4}\gamma_{4},n_{2}i_{2}\gamma_{2}}^{aa^{+}*}(\varepsilon^{\prime })+G_{n_{1}i_{1}\gamma_{1},n_{3}i_{3}\gamma_{3}}^{aa^{+}}(\varepsilon +\varepsilon^{\prime })\times \\ \times \left[G_{n_{4}i_{4}\gamma_{4},n_{2}i_{2}\gamma_{2}}^{aa^{+}}(\varepsilon^{\prime })-G_{n_{4}i_{4}\gamma_{4},n_{2}i_{2}\gamma_{2}}^{aa^{+}*}(\varepsilon^{\prime })\right]\}\times \\ \times \Gamma_{\;n_{3}i_{3}\gamma_{3},n_{4}i_{4}\gamma_{4}}^{\;n^{\prime }i^{\prime }\alpha^{\prime }}. \end{array}$$

where f(ε) is the so-called Fermi–Dirac distribution function.

**Figure 4:**
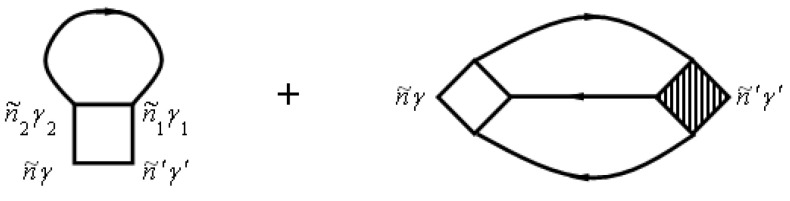
Diagrams for $\Sigma_{ee\;ni\gamma ,n^{\prime }i^{\prime }\gamma^{\prime }}(\tau ,\tau^{\prime })=\Sigma_{ee\;\tilde{n}\gamma ,{\tilde{n}}^{\prime }\gamma^{\prime }}$. Here $\tilde{n}=(ni\tau )$.

Diagrams for the mass operator $\Sigma_{ee}(\tau ,\tau^{\prime })$ that describe the electron–electron interaction, are shown in Figure 4. Diagrams for the mass operator $\Sigma_{ee}(\tau ,\tau^{\prime })$ that describe electron–electron interaction, are shown in [26,29,30].

**Figure 5:**
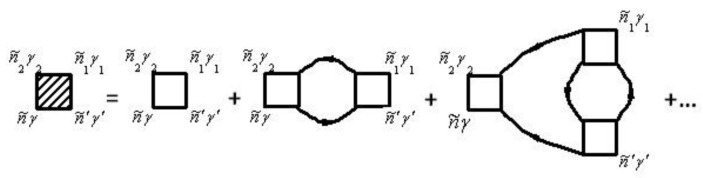
Diagrams for vertex part $\Gamma_{ni\gamma ,n^{\prime }i^{\prime }\gamma^{\prime }}^{n_{2}i_{2}\gamma_{2},n_{1}i_{1}\gamma_{1}}(\tau_{2},\tau_{1}\tau ,\tau^{\prime })=\Gamma_{\tilde{n}\gamma ,{\tilde{n}}^{\prime }\gamma^{\prime }}^{{\tilde{n}}_{2}\gamma_{2},{\tilde{n}}_{1}\gamma_{1}}$. Here $\tilde{n}=(ni\tau )$.

The vertex parts $\Gamma_{ni\gamma ,n^{\prime }i^{\prime }\gamma^{\prime }}^{n_{2}i_{2}\gamma_{2},n_{1}i_{1}\gamma_{1}}(\tau_{2},\tau_{1}\tau ,\tau^{\prime })$ are shown in diagrams in Figure 5.

Not shaded triangle in Figure 5 corresponds to equation

(74) $$\Gamma_{0\;ni\gamma ,n^{\prime }i^{\prime }\gamma^{\prime }}^{n_{2}i_{2}\gamma_{2},n_{1}i_{1}\gamma_{1}}(\tau_{2},\tau_{1}\tau ,\tau^{\prime })={\tilde{v}}_{n_{1}i_{1}\gamma_{1},n^{\prime }i^{\prime }\gamma^{\prime }}^{(2)ni\gamma ,n_{2}i_{2}\gamma_{2}}\times \delta (\tau -\tau_{2})\delta (\tau -\tau_{1})\delta (\tau -\tau^{\prime }),$$

(75) $${\tilde{v}}_{n_{1}i_{1}\gamma_{1},n^{\prime }i^{\prime }\gamma^{\prime }}^{(2)ni\gamma ,n_{2}i_{2}\gamma_{2}}=v_{n_{1}i_{1}\gamma_{1},n^{\prime }i^{\prime }\gamma^{\prime }}^{(2)ni\gamma ,n_{2}i_{2}\gamma_{2}}-v_{n^{\prime }i^{\prime }\gamma^{\prime },n_{1}i_{1}\gamma_{1}}^{(2)ni\gamma ,n_{2}i_{2}\gamma_{2}}$$

The mass operator that describes the electron–electron interaction is:

(76) $$\Sigma_{ee\;ni\gamma ,n^{\prime }i^{\prime }\gamma^{\prime }}(\varepsilon )=\Sigma_{ee\;ni\gamma ,n^{\prime }i^{\prime }\gamma^{\prime }}^{\left(1\right)}+\Sigma_{ee\;ni\gamma ,n^{\prime }i^{\prime }\gamma^{\prime }}^{\left(2\right)}(\varepsilon ),$$

(77) $$\Sigma_{\mathrm{ee}\;n,n^{\prime }}^{\left(1\right)}=-\frac{1}{2\pi i}\int_{-\infty }^{\infty }d\varepsilon^{\prime }\;f\left(\varepsilon^{\prime }\right)\;\Gamma_{\;n,\;n^{\prime }}^{\;\left(0\right)\;n_{2},n_{1}}\left[G_{n_{1},n_{2}}^{aa^{+}}(\varepsilon^{\prime })-G_{n_{1},n_{2}}^{aa^{+}\;*}(\varepsilon^{\prime })\right],$$

(78) $$\Sigma_{\mathrm{ee}\;\;n,n^{\prime }}^{\left(2\right)}\left(\varepsilon \right)={\left(\frac{1}{2\pi i}\right)}^{2}\int_{-\infty }^{\infty }d\varepsilon_{1}\int_{-\infty }^{\infty }d\varepsilon_{2}\;f\left(\varepsilon_{1}\right)f\left(\varepsilon_{2}\right)\Gamma_{\;n_{2},n_{1}}^{\;\left(0\right)\;n,n_{3}}\;\;\begin{array}{l} \times [G_{n_{2},n_{5}}^{aa^{+}\;}(\varepsilon -\varepsilon_{1}-\varepsilon_{2})G_{n_{1},n_{4}}^{aa^{+}*}(\varepsilon_{1})-\\ -G_{n_{2},n_{5}}^{aa^{+}*}(\varepsilon -\varepsilon_{1}-\varepsilon_{2})G_{n_{1},n_{4}}^{aa^{+}\;}(\varepsilon_{1})]\times \end{array}\;\begin{array}{l} \times \left[G_{n_{6},n_{3}}^{aa^{+}}\left(\varepsilon_{2}\right)-G_{n_{6},n_{3}}^{aa^{+}\;*}\left(\varepsilon_{2}\right)\right]\\ -\left[G_{n_{2},n_{5}}^{aa^{+}}\left(\varepsilon -\varepsilon_{1}-\varepsilon_{2}\right)-G_{n_{2},n_{5}}^{aa^{+}\;*}\left(\varepsilon -\varepsilon_{1}-\varepsilon_{2}\right)\right]\times \end{array}\;\begin{array}{l} \times [G_{n_{1},n_{4}}^{aa^{+}}\left(\varepsilon_{1}\right)G_{n_{6},n_{3}}^{aa^{+}}\left(\varepsilon_{2}\right)\\ -G_{n_{1},n_{4}}^{aa^{+}\;*}\left(\varepsilon_{1}\right)G_{n_{6},n_{3}}^{aa^{+}\;*}\left(\varepsilon_{2}\right)]\}\Gamma_{\;n_{4},\;n^{\prime }}^{\;n_{5},n_{6}}, \end{array}$$

(79) $$\Gamma_{n_{2},n_{1}}^{\left(0\right)n,n_{3}}={\tilde{v}}_{n_{2},n_{1}}^{(2)n,n_{3}}=v_{n_{2},n_{1}}^{(2)n,n_{3}}-v_{n_{1},n_{2}}^{(2)n,n_{3}}n=ni\gamma$$

A similar result for the contribution to the phonon self-energy $\Sigma_{phph}(\varepsilon )$ from phonon–phonon coupling is given in [26].

In deriving the expressions in Equations (72), (74), (78), and (79), we employed the standard resummation techniques for any function ϕ(z) that is analytic in the region covered by contour C, which encloses all of the Matsubara frequencies. Namely, we have

(80) $$\begin{array}{l} \Theta \sum_{\omega_{n}}\phi (i\omega_{n})=\frac{1}{4\pi i}\oint_{C}dz\;\coth\left(\frac{z}{2\Theta }\right)\phi \left(z\right)\;\\ \left(\omega_{n}=2n\pi \;\Theta \right) \end{array},$$

for the Bosonic case, and

(81) $$\begin{array}{l} \Theta \sum_{\omega_{n}}\phi (i\omega_{n})=-\frac{1}{2\pi i}\oint_{C}dz\;\tilde{f}\left(\frac{z}{\Theta }\right)\phi \left(z\right)\;\\ \left(\omega_{n}=\left(2n+1\right)\pi \;\Theta \right) \end{array},$$

for the Fermionic case, with

(82) $$\tilde{f}\left(\frac{z}{\Theta }\right)=\frac{1}{\exp\left(\frac{z}{\Theta }\right)+1}.$$

We comment that for the many-body Green’s functions described here, it is customary to have the chemical potential situated at zero frequency, as dine here.

In general, the renormalization of the vertex of the mass operator of the Green’s functions in expressions (72), (74), and (79) can be performed using Figure 2 and Figure 5. The diagrams in Figure 2 and Figure 5 correspond to the equation

(83) $$\begin{array}{l} \Gamma_{\;n_{3}i_{3}\gamma_{3},n_{4}i_{4}\gamma_{4}}^{n^{\prime }i^{\prime }\alpha^{\prime }}=\Gamma_{\;n_{3}i_{3}\gamma_{3},n_{4}i_{4}\gamma_{4}}^{\left(0\right)n^{\prime }i^{\prime }\alpha^{\prime }}-\frac{1}{2\pi i}\int_{-\infty }^{\infty }d\varepsilon f\left(\varepsilon \right)\Gamma_{\;n_{5}i_{5}\gamma_{5},n_{6}i_{6}\gamma_{6}}^{\left(0\right)n^{\prime }i^{\prime }\alpha^{\prime }}\\ \times \left[G_{n_{6}i_{6}\gamma_{6},n_{7}i_{7}\gamma_{7}}^{aa^{+}}\left(\varepsilon \right)G_{n_{8}i_{8}\gamma_{8},n_{5}i_{5}\gamma_{5}}^{aa^{+}}\left(\varepsilon \right)-G_{n_{6}i_{6}\gamma_{6},n_{7}i_{7}\gamma_{7}}^{aa^{+}*}\left(\varepsilon \right)G_{n_{8}i_{8}\gamma_{8},n_{5}i_{5}\gamma_{5}}^{aa^{+}*}\left(\varepsilon \right)\right]\\ \times \Gamma_{\;n_{7}i_{7}\gamma_{7},n_{8}i_{8}\gamma_{8}}^{\left(0\right)n_{9}i_{9}\alpha_{9}}G_{n_{9}i_{9}\alpha_{9},n_{10}i_{10}\alpha_{10}}^{uu}\left(0\right)\Gamma_{n_{3}i_{3}\gamma_{3},n_{4}i_{4}\gamma_{4}}^{n_{10}i_{10}\alpha_{10}} \end{array}$$

and

(84) $$\begin{array}{l} \Gamma_{\;n_{4},\;n^{\prime }}^{\;n_{5},n_{6}}=\Gamma_{\;n_{4},\;n^{\prime }}^{\left(0\right)\;n_{5},n_{6}}-\frac{1}{2\pi i}\int_{-\infty }^{\infty }d\varepsilon f\left(\varepsilon \right)\Gamma_{\;n_{4},\;n_{8}}^{\left(0\right)\;n_{5},n_{7}}\\ \times \left[G_{n_{7},n_{9}}^{aa^{+}}\left(\varepsilon \right)G_{n_{8},n_{10}}^{aa^{+}*}\left(\varepsilon \right)-G_{n_{7},n_{9}}^{aa^{+}*}\left(\varepsilon \right)G_{n_{8},n_{10}}^{aa^{+}}\left(\varepsilon \right)\right]\\ \times \Gamma_{\;n_{10},\;n^{\prime }}^{\;n_{9},n_{6}},n=ni\gamma . \end{array}$$

Summation is implied over repeated indices in expressions (84) and (85).

The Fermi level $\varepsilon_{F}\equiv \mu_{e}$ of the system is determined by equation:

(85) $$<Z>=\int_{-\infty }^{\infty }f(\varepsilon )\;g_{e}(\varepsilon )\;d\varepsilon ,$$

(86) $$f(\varepsilon )=\frac{1}{\exp(\frac{\varepsilon -\varepsilon_{F}}{\Theta }\;)+1}$$

where <Z> is the average number of electrons per atom and $g_{e}(\varepsilon )$ is the many-body electronic density of states, which satisfies

(87) ge(ε)=−1πνN Im Tr〈Gaa+(ε)〉c

Here, ${\langle \ldots \rangle }_{c}$ denotes configurational averaging over the disorder, *N* is the number of primitive lattice cells, and ν is the number of atoms per primitive cell. We drop the letter *c* on the configurational averaging for simplicity. In Equation (86), 〈Z〉 is the average number of electrons per atom.

It should be noted that the first term in the electron self-energy due to electron–electron interactions, $\Sigma_{ee\;ni\gamma ,n^{\prime }i^{\prime }\gamma^{\prime }}^{\left(1\right)}$ in Equation (77), describes the Coulomb and exchange electron–electron interactions in the Hartree–Fock approximation. The second term, $\Sigma_{ee\;ni\gamma ,n^{\prime }i^{\prime }\gamma^{\prime }}^{\left(2\right)}(\varepsilon )$, which is caused by corrections beyond Hartree–Fock, describes the effects of electron correlations. As opposed to the procedures used in [13,14,15,16,17,18,19], the long-range Coulomb interaction of electrons located at different lattice sites of the crystal is described by taking into account an arbitrary number of energy bands.

The expression for the Green’s function in Equations (67) and (68) differs from the corresponding expressions for the Green’s function of a single-particle Hamiltonian of a disordered system, only from the different self-energy contributions. Hence, we solve for the Green’s function using the well-known methods of disordered systems theory [31,32].

To find the density of states by Formula (88), it is necessary to find the average values of the Green’s functions defined by Formulas (67) and (68).

## 4. Localized Magnetic Moments

As we will be working with magnetic moments in the remainder of the paper, we now slightly modify our notation so that the symbol $\gamma =\delta \sigma =\tilde{\varepsilon }lm\sigma$ now refers to all other quantum numbers, except for spin, and we introduce the spin quantum number σ explicitly in all of the following equations. The electron–electron self-energy in Equation (67) requires the occupation number $Z_{ni\delta \sigma }^{\;\lambda m_{\lambda i}}$ of the different electronic states (niδσ), where we are explicitly including the dependence on σ. The explicit values for $Z_{ni\delta \sigma }^{\;\lambda m_{\lambda i}}$ are calculated from Equation (86), where the total electronic density of states $g_{e}(\varepsilon )$ is replaced by the partial density of states $g_{ni\delta \sigma }^{\lambda \;m_{\lambda i}}(\varepsilon )$ for energy band δ and spin projection σ, to allow for magnetic solutions. The occupation numbers $Z_{ni\delta \sigma }^{\;\lambda m_{\lambda i}}$ and the partial density of states $g_{ni\delta \sigma }^{\lambda \;m_{\lambda i}}(\varepsilon )$ then satisfy

(88) Zniδσ λmλi=∫−∞∞f(ε) gniδσλmλi(ε) dε,

(89) gniδσλmλi(ε)=−1πIm〈Gniδσ,niδσaa+(ε)〉|(ni)∈λmλi

Note that disorder averaging is done under the assumption that an atom of type λ is located at the site (ni), and its projection of the localized magnetic moment onto the *z*-axis is equal to $m_{\lambda i}$. The probability of this configuration is $P_{ni}^{\lambda m_{\lambda i}}$, and we have the obvious constraint that

(90) $$\sum_{\lambda ,m_{\lambda i}}P_{ni}^{\lambda m_{\lambda i}}=1.\;$$

The total charge and magnetization for each orbital on a site are given by

(91) $$Z_{ni\delta }^{\lambda m_{\lambda i}}=Z_{ni\delta \sigma }^{\lambda m_{\lambda i}}+Z_{ni\delta ,-\sigma }^{\lambda m_{\lambda i}},\;m_{\lambda i\delta }=Z_{ni\delta \sigma }^{\lambda m_{\lambda i}}-Z_{ni\delta ,-\sigma }^{\lambda m_{\lambda i}},$$

and by

(92) $$Z_{ni\delta \sigma }^{\lambda m_{\lambda i}}=\frac{Z_{ni\delta }^{\lambda m_{\lambda i}}+m_{\lambda i\delta }}{2},\;Z_{ni\delta ,-\sigma }^{\lambda m_{\lambda i}}=\frac{Z_{ni\delta }^{\lambda m_{\lambda i}}-m_{\lambda i\delta }}{2},$$

respectively. We need to sum over all other quantum numbers to get the totals:

(93) $$Z_{ni}^{\lambda m_{\lambda i}}=\sum_{\delta }Z_{ni\delta }^{\lambda m_{\lambda i}},\;m_{\lambda i}=\sum_{\delta }m_{\lambda i\delta }.$$

## 5. Density of Electronic and Phononic States

In Equations (67) and (68), by introducing the mass operator as the sum of one site operators and selecting it as a zero approximation, the effective medium Green’s function cluster expansion for Green’s functions $G^{aa^{+}}(\varepsilon )$, $G^{uu}(\varepsilon )$, is performed. The specified expansion is a generalization of the cluster expansion for the Green’s function $G^{aa^{+}}(\varepsilon )$ of single-particle Hamiltonian. Green’s functions of the effective environment are defined by the expressions:

(94) $${\tilde{G}}^{aa^{+}}(\varepsilon )={\left[\varepsilon -h^{\left(0\right)}-{\tilde{\Sigma }}_{eph}(\varepsilon )-{\tilde{\Sigma }}_{ee}(\varepsilon )-\sigma_{e}\left(\varepsilon \right)\right]}^{-1},$$

(95) $${\tilde{G}}^{uu}(\varepsilon )={\left[\frac{\varepsilon^{2}}{\hbar^{2}}M^{\left(0\right)}-\Phi^{\left(0\right)}-{\tilde{\Sigma }}_{phe}(\varepsilon )-\sigma_{ph}\left(\varepsilon \right)\right]}^{-1}.$$

Expressions for the operators ${\tilde{\Sigma }}_{eph}(\varepsilon )$, ${\tilde{\Sigma }}_{phe}(\varepsilon )$, and ${\tilde{\Sigma }}_{ee}(\varepsilon )$ are obtained from the expressions for the mass operators $\Sigma_{eph}(\varepsilon )$, $\Sigma_{phe}(\varepsilon )$, and $\Sigma_{ee}(\varepsilon )$ (72)–(80) by replacing the vertex parts $\Gamma_{\;ni\gamma ,\;n_{3}i_{3}\gamma_{3}}^{\left(0\right)n_{1}i_{1}\alpha_{1}}$, $\Gamma_{n_{2},n_{1}}^{\left(0\right)n,n_{3}}$, n≡niγ by their expressions for ideally ordered crystals, and replacing the Green’s functions $G^{aa^{+}}(\varepsilon )$, $G^{uu}(\varepsilon )$ with the Green’s functions of the effective medium ${\tilde{G}}^{aa^{+}}(\varepsilon )$, ${\tilde{G}}^{uu}(\varepsilon )$. Expressions for operators $\sigma_{e}\left(\varepsilon \right)$ and $\sigma_{ph}\left(\varepsilon \right)$ in Formulas (95) and (96) will be given below.

The Green’s functions in Equations (67) and (68) satisfy a Dyson equation that can be expressed in terms of a T-matrix via:

(96) $$G(\varepsilon )=\tilde{G}(\varepsilon )+\tilde{G}(\varepsilon )\;T(\varepsilon )\;\tilde{G}(\varepsilon ),$$

where the T-matrix *T* is represented by a series, in which each term describes the scattering of clusters with different numbers of nodes expressed schematically by

(97) $$T=\sum_{\left(n_{1}i_{1}\right)}t^{n_{1}i_{1}}+\sum_{\left(n_{1}i_{1})\neq (n_{2}i_{2}\right)}^{}T^{(2)\;n_{1}i_{1},n_{2}i_{2}}+\ldots$$

Here, we have

(98) $$T^{(2)\;n_{1}i_{1},n_{2}i_{2}}={\left[I-t^{n_{1}i_{1}}\tilde{G}t^{n_{2}i_{2}}\tilde{G}\right]}^{-1}t^{n_{1}i_{1}}\tilde{G}t^{n_{2}i_{2}}\left[I+\tilde{G}t^{n_{1}i_{1}}\right],$$

where $t^{n_{1}i_{1}}$ is the on-site scattering operator, which is given by

(99) $$t^{n_{1}i_{1}}={\left[I-(\Sigma^{n_{1}i_{1}}-\sigma^{n_{1}i_{1}})\tilde{G}\right]}^{-1}(\Sigma^{n_{1}i_{1}}-\sigma^{n_{1}i_{1}}).$$

The self-energy employed in Equation (67), $\Sigma_{e}^{n_{1}i_{1}}(\varepsilon )$, satisfies

(100) $$w+\Sigma_{eph}(\varepsilon )+\Sigma_{ee}(\varepsilon )-{\tilde{\Sigma }}_{eph}(\varepsilon )-{\tilde{\Sigma }}_{ee}(\varepsilon )=\sum_{\left(n_{1}i_{1}\right)}\Sigma_{e}^{n_{1}i_{1}}(\varepsilon ),$$

for the electrons. For the phonons, we have

(101) $$\begin{array}{l} \frac{\varepsilon^{2}}{\hbar^{2}}\Delta M+\Delta \Phi +\Sigma_{phe}(\varepsilon )+\Sigma_{phph}(\varepsilon )-\\ -{\tilde{\Sigma }}_{phe}(\varepsilon )-{\tilde{\Sigma }}_{phph}(\varepsilon )=\sum_{\left(n_{1}i_{1}\right)}\Sigma_{ph}^{n_{1}i_{1}}(\varepsilon ) \end{array}$$

Using Equations (72), (77)–(79), and (101), we obtain the expression for the intrinsic energy part $\Sigma_{eni\gamma ,n^{\prime }i^{\prime }\gamma^{\prime }}^{\lambda n_{1}i_{1}}(\varepsilon )$, which describes the scattering of electrons:

(102) $$\begin{array}{l} \Sigma_{en_{1}i_{1}\gamma_{1},n_{2}i_{2}\gamma_{2}}^{\lambda m_{\lambda i}ni}(\varepsilon )=w_{n_{1}i_{1}\gamma_{1},n_{2}i_{2}\gamma_{2}}^{\lambda ni}+\\ +\sum_{\begin{array}{l} n_{3}i_{3}\gamma_{3}\\ n_{4}i_{4}\gamma_{4} \end{array}}{\tilde{v}}_{n_{3}i_{3}\gamma_{3},n_{2}i_{2}\gamma_{2}}^{(2)n_{1}i_{1}\gamma_{1},n_{4}i_{4}\gamma_{4}}(Z_{n_{3}i_{3}\gamma_{3},n_{4}i_{4}\gamma_{4}}^{\lambda m_{\lambda i}ni}-{\tilde{Z}}_{n_{3}i_{3}\gamma_{3},n_{4}i_{4}\gamma_{4}}^{\lambda m_{\lambda i}ni}), \end{array}$$

where

(103) Zn3i3γ3,n4i4γ4λmλini=−1π∫−∞∞f(ε,εF) Im〈Gn3i3γ3,n4i4γ4aa+(ε)〉|(ni)∈λmλi dε.

The value of ${\tilde{Z}}_{n_{3}i_{3}\gamma_{3},n_{4}i_{4}\gamma_{4}}^{\lambda m_{\lambda i}ni}$ in Equation (103) is derived from Equation (104) by replacing the full Green’s function by the effective medium Green’s function. The diagonal elements of the matrix $Z_{n_{3}i_{3}\gamma_{3},n_{4}i_{4}\gamma_{4}}^{\lambda m_{\lambda i}ni}$ in Equation (104) are equal to the occupation numbers of the electron states $Z_{ni\delta \sigma }^{\;\lambda m_{\lambda i}}$ in Equation (89).

Using Equations (10), (70), (74), and (102), we obtain the expression for the intrinsic energy part $\Sigma_{phni\alpha ,n^{\prime }i^{\prime }\alpha^{\prime }}^{\lambda n_{1}i_{1}}(\varepsilon )$, which describes the scattering of phonons:

(104) $$\Sigma_{phni\alpha ,n^{\prime }i^{\prime }\alpha^{\prime }}^{\lambda n_{1}i_{1}}(\varepsilon )=\frac{\varepsilon^{2}}{\hbar^{2}}(M_{i_{1}}-M_{\lambda })\delta_{nn^{\prime }}\delta_{ii^{\prime }}\delta_{\alpha \alpha^{\prime }}$$

It should be noted that, in the limit of an infinite crystal, on the right-hand side of Equations (103) and (105), the terms inversely proportional to the number of lattice sites are neglected.

We require the fulfillment of the condition

(105) $$\langle t^{0i_{1}}\rangle =0,$$

from which follows the system of coupled equations for the operator, in Formulas (95) and (96):

(106) $$\begin{array}{l} \sigma_{e}^{0i_{1}}(\varepsilon )={\langle {\left[1-(\Sigma_{e}^{0i_{1}}(\varepsilon )-\sigma_{e}^{0i_{1}}(\varepsilon )){\tilde{G}}_{}^{aa^{+}}(\varepsilon )\right]}^{-1}\rangle }^{-1}\times \\ \times \langle {\left[1-(\Sigma_{e}^{0i_{1}}(\varepsilon )-\sigma_{e}^{0i_{1}}(\varepsilon )){\tilde{G}}_{}^{aa^{+}}(\varepsilon )\right]}^{-1}\Sigma_{e}^{0i_{1}}(\varepsilon )\rangle \end{array},$$

(107) $$\begin{array}{l} \sigma_{ph}^{0i_{1}}(\varepsilon )={\langle {\left[1-(\Sigma_{ph}^{0i_{1}}(\varepsilon )-\sigma_{ph}^{0i_{1}}(\varepsilon )){\tilde{G}}_{}^{uu}(\varepsilon )\right]}^{-1}\rangle }^{-1}\times \\ \times \langle {\left[1-(\Sigma_{ph}^{0i_{1}}(\varepsilon )-\sigma_{ph}^{0i_{1}}(\varepsilon )){\tilde{G}}_{}^{uu}(\varepsilon )\right]}^{-1}\Sigma_{ph}^{0i_{1}}(\varepsilon )\rangle \end{array}.$$

The matrix elements of the Green’s function of the electron subsystem of the effective medium can be calculated using Fourier transformation

(108) $${\tilde{G}}_{ni\gamma ,n^{\prime }i^{\prime }\gamma^{\prime }}^{aa^{+}}(\varepsilon )=\frac{1}{N}\sum_{k}{\tilde{G}}_{i\gamma ,i^{\prime }\gamma^{\prime }}^{aa^{+}}(k,\varepsilon )e^{ik(r_{ni}-r_{n^{\prime }i^{\prime }})},$$

(109) $${\tilde{G}}^{aa^{+}}(k,\varepsilon )={\left(\varepsilon -\tilde{H}\left(k,\varepsilon \right)\right)}^{-1},$$

where

(110) $$\tilde{H}\left(k,\varepsilon \right)=h^{\left(0\right)}\left(k\right)+{\tilde{\Sigma }}_{eph}\left(k,\varepsilon \right)+{\tilde{\Sigma }}_{ee}\left(k,\varepsilon \right)+\sigma_{e}\left(k,\varepsilon \right),$$

N is the number of primitive unit cells.

We do a similar procedure for the effective medium phonon Green’s function, which satisfies

(111) $${\tilde{G}}_{ni\alpha ,n^{\prime }i^{\prime }\alpha^{\prime }}^{uu}(\varepsilon )=\frac{1}{N}\sum_{k}{\tilde{G}}_{i\alpha ,i^{\prime }\alpha^{\prime }}^{uu}(k,\varepsilon )e^{ik(r_{ni}-r_{n^{\prime }i^{\prime }})},$$

(112) $${\tilde{G}}^{uu}(k,\varepsilon )={\left(\frac{\varepsilon^{2}}{\hbar^{2}}M^{\left(0\right)}-\tilde{\Phi }\left(k,\varepsilon \right)\right)}^{-1},$$

where we have

(113) $$\tilde{\Phi }\left(k,\varepsilon \right)=\Phi^{\left(0\right)}\left(k\right)+{\tilde{\Sigma }}_{phe}\left(k,\varepsilon \right)+{\tilde{\Sigma }}_{phph}\left(k,\varepsilon \right)+\sigma_{ph}\left(\varepsilon \right),$$

(114) $$M_{i\alpha ,i^{\prime }\alpha^{\prime }}^{\left(0\right)}=M_{i}\delta_{ii^{\prime }}\delta_{\alpha \alpha^{\prime }}.$$

Note that wave vector k varies within the first Brillouin zone. Furthermore, we have that ${\tilde{\Sigma }}_{eph}\left(k,\varepsilon \right)$ is the Fourier transformation of the matrix $\Sigma_{eph\;ni\gamma ,n^{\prime }i^{\prime }\gamma^{\prime }}(\varepsilon )$ given in Equation (72), for which the terms $v^{\prime }{}_{ni\gamma ,\;n_{3}i_{3}\gamma_{3}}^{n_{1}i_{1}\alpha_{1}}$ are replaced by the values for a pure crystal and the corresponding Green’s functions are those of the effective medium. The other self-energies given by ${\tilde{\Sigma }}_{ee}\left(k,\varepsilon \right)$, ${\tilde{\Sigma }}_{phe}\left(k,\varepsilon \right)$, and ${\tilde{\Sigma }}_{phph}\left(k,\varepsilon \right)$ are defined similarly. In Equation (114), $\Phi^{\left(0\right)}\left(k\right)$ is the Fourier transform of the matrix $\Phi^{\left(0\right)}{}_{ni\alpha ,n^{\prime }i^{\prime }\alpha^{\prime }}$, which describes the atomic nucleus repulsion. The self-energy ${\tilde{\Sigma }}_{phe}\left(k,\varepsilon \right)$ describes the attractive interaction between the atomic nuclei and the electrons.

The matrix $\Sigma_{phni\alpha ,n^{\prime }i^{\prime }\alpha^{\prime }}^{\lambda n_{1}i_{1}}(\varepsilon )$ in expression (105) is diagonal. From expression (108), it follows that the matrix $\sigma_{ph}\left(\varepsilon \right)$ is also diagonal, and its Fourier transform $\sigma_{phi\alpha ,i^{\prime }\alpha^{\prime }}\left(\varepsilon \right)=\sigma_{phi}\left(\varepsilon \right)\delta_{ii^{\prime }}\delta_{\alpha \alpha^{\prime }}$ in expression (114) does not depend on the wave vector k. In the diagonal disorder approximation, the matrix $w_{ni\gamma ,n^{\prime }i^{\prime }\gamma^{\prime }}^{\lambda n_{1}i_{1}}$ in expression (103) is diagonal in indices $n,n^{\prime }$. Neglecting the second term on the right-hand side of Equation (103), we obtain from Equation (107) that in this approximation the matrix $\sigma_{e}\left(\varepsilon \right)$ is also diagonal in indices $n,n^{\prime }$, and its Fourier transform $\sigma_{ei\gamma ,i^{\prime }\gamma^{\prime }}\left(\varepsilon \right)$ in expression (111) does not depend on wave vector k. The Fourier transform of the mass operator of electron–phonon interaction has the form:

(115) $$\begin{array}{l} {\tilde{\Sigma }}_{eph\;i\gamma ,i^{\prime }\gamma^{\prime }}(k,\varepsilon )=-\frac{1}{4\pi i}\frac{1}{N}\int_{-\infty }^{\infty }d\varepsilon_{1}\cot h\left(\frac{\varepsilon_{1}}{2\Theta }\right)\\ \times \sum_{k_{1}}\;\Gamma_{\;i\gamma ,\;i_{3}\gamma_{3}}^{\left(0\right)i_{1}\alpha_{1}}\left(-k,k-k_{1}\right)\left[{\tilde{G}}_{i_{1}\alpha_{1},i_{2}\alpha_{2}}^{uu}(k_{1}\varepsilon_{1})-{\tilde{G}}_{i_{1}\alpha_{1},i_{2}\alpha_{2}}^{uu\;*}(k_{1}\varepsilon_{1})\right]\\ \times {\tilde{G}}_{i_{3}\gamma_{3},i_{4}\gamma_{4}}^{aa^{+}}\left(k-k_{1},\varepsilon -\varepsilon_{1}\right){\tilde{\Gamma }}_{\;i_{4}\gamma_{4},i^{\prime }\gamma^{\prime }}^{i_{2}\alpha_{2}}\left(-k+k_{1},k\right). \end{array}$$

The Fourier transform of the phonon–electron interaction mass operator is:

(116) $$\begin{array}{l} {\tilde{\Sigma }}_{phe\;ni\alpha ,n^{\prime }i^{\prime }\alpha^{\prime }}(k,\varepsilon )=\frac{1}{2\pi i}\frac{1}{N}\int_{-\infty }^{\infty }d\varepsilon_{1}\;f(\varepsilon_{1})\sum_{}\Gamma_{i_{2}\gamma_{2},i_{1}\gamma_{1}}^{\;\left(0\right)i\alpha }\left(-k_{1},k+k_{1}\right)\\ \times \{\left[{\tilde{G}}_{i_{1}\gamma_{1},i_{3}\gamma_{3}}^{aa^{+}}(k+k_{1},\varepsilon +\varepsilon_{1})-{\tilde{G}}_{i_{1}\gamma_{1},i_{3}\gamma_{3}}^{aa^{+}*}(k+k_{1},\varepsilon +\varepsilon_{1})\right]\\ \times {\tilde{G}}_{i_{4}\gamma_{4},i_{2}\gamma_{2}}^{aa^{+}*}(k_{1},\varepsilon_{1})+{\tilde{G}}_{i_{1}\gamma_{1},i_{3}\gamma_{3}}^{aa^{+}}(k+k_{1},\varepsilon +\varepsilon_{1})\\ \times \left[{\tilde{G}}_{i_{4}\gamma_{4},i_{2}\gamma_{2}}^{aa^{+}}(k_{1},\varepsilon_{1})-{\tilde{G}}_{i_{4}\gamma_{4},i_{2}\gamma_{2}}^{aa^{+}*}(k_{1},\varepsilon_{1})\right]\}{\tilde{\Gamma }}_{\;i_{3}\gamma_{3},i_{4}\gamma_{4}}^{\;i^{\prime }\alpha^{\prime }}\left(-k-k_{1},k_{1}\right). \end{array}$$

The vertex parts of the mass operators of electron–phonon and phonon–electron interactions are determined by the equation:

(117) $$\begin{array}{l} {\tilde{\Gamma }}_{\;i_{3}\gamma_{3},i_{4}\gamma_{4}}^{ni^{\prime }\alpha^{\prime }}\left(k_{1},k_{2}\right)=\Gamma_{\;i_{3}\gamma_{3},i_{4}\gamma_{4}}^{\left(0\right)i^{\prime }\alpha^{\prime }}\left(k_{1},k_{2}\right)-\frac{1}{2\pi i}\frac{1}{N}\int_{-\infty }^{\infty }d\varepsilon f\left(\varepsilon \right)\\ \times \sum_{}\Gamma_{\;i_{5}\gamma_{5},i_{6}\gamma_{6}}^{\left(0\right)i^{\prime }\alpha^{\prime }}\left(k_{1}+k_{2}-k_{5},k_{5}\right)\\ \times [{\tilde{G}}_{i_{6}\gamma_{6},i_{7}\gamma_{7}}^{aa^{+}}\left(k_{5},\varepsilon \right){\tilde{G}}_{i_{8}\gamma_{8},i_{5}\gamma_{5}}^{aa^{+}}\left(-k_{1}-k_{2}+k_{5}\varepsilon \right)\\ -{\tilde{G}}_{i_{6}\gamma_{6},i_{7}\gamma_{7}}^{aa^{+}*}\left(k_{5},\varepsilon \right){\tilde{G}}_{i_{8}\gamma_{8},i_{5}\gamma_{5}}^{aa^{+}*}\left(-k_{1}-k_{2}+k_{5}\varepsilon \right)]\\ \times \Gamma_{\;i_{7}\gamma_{7},i_{8}\gamma_{8}}^{\left(0\right)i_{9}\alpha_{9}}\left(-k_{5},-k_{1}-k_{2}+k_{5}\right){\tilde{G}}_{i_{9}\alpha_{9},i_{10}\alpha_{10}}^{uu}\left(k_{1}+k_{2},0\right)\\ \times {\tilde{\Gamma }}_{i_{3}\gamma_{3},i_{4}\gamma_{4}}^{i_{10}\alpha_{10}}\left(k_{1},k_{1}+k_{2}\right). \end{array}$$

In expressions (116)–(118)

(118) $$\begin{array}{l} \Gamma_{\;i_{1}\gamma_{1},i_{2}\gamma_{2}}^{\left(0\right)i\alpha }\left(k_{1},k_{2}\right)=\\ \sum_{n_{1},n_{2}}v^{\prime }{}_{n_{1}i_{1}\gamma_{1},n_{2}i_{2}\gamma_{2}}^{ni\alpha }\exp\left(ik_{1}\left(r_{n_{1}i_{1}}-r_{ni}\right)+ik_{2}\left(r_{n_{2}i_{2}}-r_{ni}\right)\right). \end{array}$$

The Fourier transform of the mass operator of the electron–electron interaction can be represented as:

(119) $${\tilde{\Sigma }}_{ee\;i\gamma ,i^{\prime }\gamma^{\prime }}(k,\varepsilon )={\tilde{\Sigma }}_{ee\;i\gamma ,i^{\prime }\gamma^{\prime }}^{\left(1\right)}\left(k\right)+{\tilde{\Sigma }}_{ee\;i\gamma ,i^{\prime }\gamma^{\prime }}^{\left(2\right)}(k,\varepsilon ),$$

(120) $$\begin{array}{l} {\tilde{\Sigma }}_{\mathrm{ee}\;i\gamma ,i^{\prime }\gamma^{\prime }}^{\left(1\right)}\left(k\right)=-\frac{1}{2\pi i}\frac{1}{N}\\ \times \int_{-\infty }^{\infty }d\varepsilon_{1}\;f\left(\varepsilon_{1}\right)\sum_{k_{1}}\Gamma_{i\gamma ,i^{\prime }\gamma^{\prime }}^{\;\left(0\right)\;i_{2}\gamma_{2},i_{1}\gamma_{1}}\left(-k,-k_{1},k_{1}\right)\\ \times \left[{\tilde{G}}_{i_{1}\gamma_{1},i_{2}\gamma_{2}}^{aa^{+}}(k_{1},\varepsilon_{1})-{\tilde{G}}_{i_{1}\gamma_{1},i_{2}\gamma_{2}}^{aa^{+}\;*}(k_{1},\varepsilon_{1})\right] \end{array},$$

(121) $$\begin{array}{l} {\tilde{\Sigma }}_{\mathrm{ee}\;i\gamma ,i^{\prime }\gamma^{\prime }}^{\left(2\right)}\left(k,\varepsilon \right)={\left(\frac{1}{2\pi i}\right)}^{2}\frac{1}{N^{2}}\int_{-\infty }^{\infty }d\varepsilon_{1}\int_{-\infty }^{\infty }d\varepsilon_{2}\\ \times f\left(\varepsilon_{1}\right)f\left(\varepsilon_{2}\right)\sum_{k_{1},k_{2}}\Gamma_{{\;i}_{2}\gamma_{2},i_{1}\gamma_{1}}^{\;\left(0\right)\;i\gamma ,i_{3}\gamma_{3}}\left(-k,-k_{1}-k_{2}+k,k_{1}\right) \end{array}\;\begin{array}{l} \times \{[G_{i_{2}\gamma_{2},i_{5}\gamma_{5}}^{aa^{+}\;}(k-k_{1}-k_{2},\varepsilon -\varepsilon_{1}-\varepsilon_{2})G_{i_{1}\gamma_{1},i_{4}\gamma_{4}}^{aa^{+}*}(k_{1},\varepsilon_{1})-\\ -G_{i_{2}\gamma_{2},i_{5}\gamma_{5}}^{aa^{+}*\;}(k-k_{1}-k_{2},\varepsilon -\varepsilon_{1}-\varepsilon_{2})G_{i_{1}\gamma_{1},i_{4}\gamma_{4}}^{aa^{+}}(k_{1},\varepsilon_{1})] \end{array}\;\begin{array}{l} \times \left[{\tilde{G}}_{i_{6}\gamma_{6},i_{3}\gamma_{3}}^{aa^{+}}\left(k_{2},\varepsilon_{2}\right)-{\tilde{G}}_{i_{6}\gamma_{6},i_{3}\gamma_{3}}^{aa^{+}*}\left(k_{2},\varepsilon_{2}\right)\right]\\ -[{\tilde{G}}_{i_{2}\gamma_{2},i_{5}\gamma_{5}}^{aa^{+}}\left(k-k_{1}-k_{2},\varepsilon -\varepsilon_{1}-\varepsilon_{2}\right)\\ -{\tilde{G}}_{i_{2}\gamma_{2},i_{5}\gamma_{5}}^{aa^{+}*}\left(k-k_{1}-k_{2},\varepsilon -\varepsilon_{1}-\varepsilon_{2}\right)] \end{array}\;\begin{array}{l} \times [{\tilde{G}}_{i_{1}\gamma_{1},i_{4}\gamma_{4}}^{aa^{+}}\left(k_{1},\varepsilon_{1}\right){\tilde{G}}_{i_{6}\gamma_{6},i_{3}\gamma_{3}}^{aa^{+}}\left(k_{2},\varepsilon_{2}\right)\\ -{\tilde{G}}_{i_{1}\gamma_{1},i_{4}\gamma_{4}}^{aa^{+}*}\left(k_{1},\varepsilon_{1}\right){\tilde{G}}_{i_{6}\gamma_{6},i_{3}\gamma_{3}}^{aa^{+}*}\left(k_{2},\varepsilon_{2}\right)]\}\\ \times {\tilde{\Gamma }}_{\;i_{4}\gamma_{4},i^{\prime }\gamma^{\prime }}^{\;i_{5}\gamma_{5},i_{6}\gamma_{6}}\left(k_{1}+k_{2}-k,-k_{2},k_{1}\right). \end{array}$$

The vertex part of the mass operator of the electron–electron interaction is determined by the equation:

(122) $$\begin{array}{l} {\tilde{\Gamma }}_{\;i_{4}\gamma_{4},i^{\prime }\gamma^{\prime }}^{\;i_{5}\gamma_{5},i_{6}\gamma_{6}}\left(k_{1},k_{2},k_{3}\right)=\Gamma_{\;i_{4}\gamma_{4},i^{\prime }\gamma^{\prime }}^{\;\left(0\right)i_{5}\gamma_{5},i_{6}\gamma_{6}}\left(k_{1},k_{2},k_{3}\right)\\ -\frac{1}{2\pi i}\frac{1}{N}\int_{-\infty }^{\infty }d\varepsilon f\left(\varepsilon \right)\sum_{k_{4}}\Gamma_{{\;i}_{4}\gamma_{4},i_{8}\gamma_{8}}^{\left(0\right)\;i_{5}\gamma_{5},i_{7}\gamma_{7}}\left(k_{1},k_{2},k_{4}\right)\\ \times [{\tilde{G}}_{i_{7}\gamma_{7},i_{9}\gamma_{9}}^{aa^{+}}\left(k_{4},\varepsilon \right){\tilde{G}}_{i_{8}\gamma_{8},i_{10}\gamma_{10}}^{aa^{+}*}\left(-k_{1}-k_{2}-k_{4},\varepsilon \right)\\ -{\tilde{G}}_{i_{7}\gamma_{7},i_{9}\gamma_{9}}^{aa^{+}*}\left(k_{4},\varepsilon \right){\tilde{G}}_{i_{8}\gamma_{8},i_{10}\gamma_{10}}^{aa^{+}}\left(-k_{1}-k_{2}-k_{4},\varepsilon \right)]\\ \times {\tilde{\Gamma }}_{\;i_{10}\gamma_{10},i^{\prime }\gamma^{\prime }}^{\;i_{9}\gamma_{9},i_{6}\gamma_{6}}\left(k_{1}+k_{2}+k_{4},-k_{4},k_{3}\right). \end{array}$$

In expression (123)

(123) $$\begin{array}{l} \Gamma_{\;i_{1}\gamma_{1},i\gamma }^{\left(0\right)i_{2}\gamma_{2},i_{3}\gamma_{3}}\left(k_{1},k_{2},k_{3}\right)=\\ \sum_{n_{1},n_{2},n_{3}}{\tilde{v}}_{n_{3}i_{3}\gamma_{3},ni\gamma }^{\left(2\right)\;n_{1}i_{1}\gamma_{1},n_{2}i_{2}\gamma_{2}}\exp\left(ik_{1}\left(r_{n_{1}i_{1}}-r_{ni}\right)\right)\\ \times \exp\left(ik_{2}\left(r_{n_{2}i_{2}}-r_{ni}\right)+ik_{3}\left(r_{n_{3}i_{3}}-r_{ni}\right)\right). \end{array}$$

Cluster decomposition for the Green’s function of electrons and phonons of disordered crystal can be obtained from Equations (95)–(100). The density of the electron and phonon states are presented as infinite series. Here, processes of scattering on clusters with different numbers of atoms are described by each term. It is shown that the contribution of the scattering processes of electrons and phonons in clusters decreases with increasing the number of atoms in the cluster by a small parameter

(124) $$p(\varepsilon )=\frac{1}{r\nu }\left|\sum_{(n_{2}i_{2})\neq (n_{1}i_{1}),i,\gamma }{\langle t^{n_{1}i_{1}}(\varepsilon )\tilde{G}(\varepsilon )t^{n_{2}i_{2}}(\varepsilon )\tilde{G}(\varepsilon )\rangle }_{0i\gamma ,0i\gamma }\right|,$$

where r is the total number of energy bands included in the calculation.

We have shown previously [26,30,31,32] that this parameter remains small when many parameters of the system are changed, except possibly for narrow energy intervals near the band edges.

By neglecting the contribution of processes of electron scattering in clusters consisting of three or more atoms that are small by the above parameter in Equation (125) for the density of electronic states, we obtain:

(125) $$g_{e}\left(\varepsilon \right)=\frac{1}{v}\sum_{i,\delta ,\sigma ,\lambda ,m_{\lambda i}}P_{0i}^{\lambda m_{\lambda i}}g_{0\;i\delta \;\sigma }^{\lambda m_{\lambda i}}\left(\varepsilon \right),$$

(126) g0 iδ σλmλi(ε)=−1π Im {G˜+G˜ tλmλi0i G˜+∑(lj)≠(0i)λ′,mλ′jP        lj  0 iλ′ mλ′j/λ mλi×G˜ [tλ′mλ′jlj +T(2)λmλi 0i,λ′mλ′jlj]G˜ }0iδσ,0iδσ,

(127) $$\begin{array}{l} T^{(2)\lambda m_{\lambda i}\;0i,\lambda^{\prime }m_{\lambda^{\prime }j}lj}={\left[I-t^{\lambda m_{\lambda i}\;0i}\tilde{G}t^{\lambda^{\prime }m_{\lambda^{\prime }j}lj}\tilde{G}\right]}^{-1}\\ \times t^{\lambda m_{\lambda i}\;0i}\tilde{G}t^{\lambda^{\prime }m_{\lambda^{\prime }j}lj}\left[I+\tilde{G}t^{\lambda m_{\lambda i}\;0i}\right] \end{array}$$

where $\tilde{G}={\tilde{G}}^{aa^{+}}(\varepsilon )$.

Similarly averaging of the phonon Green’s function $G^{uu}(\varepsilon )$ yields the phononic density of states:

(128) $$g_{ph}\left(\varepsilon \right)=\frac{1}{\nu }\sum_{i,\alpha ,\lambda }P_{0i}^{\lambda }g_{0i\alpha \;}^{\lambda }\left(\varepsilon \right),$$

(129) g 0iαλ(ε)=−1π 2εℏ2MiIm {G˜+G˜ tλ 0i G˜+∑(lj)≠(0i)λ′P        lj   0iλ′/λ ×G˜ [tλ′ lj +T(2)λ 0i,λ′lj]G˜ }0iα,0iα,

where $\tilde{G}={\tilde{G}}^{uu}(\varepsilon )$.

In Equation (127), $P_{\;\;\;\;\;\;\;\;lj\;\;0\;i}^{\lambda^{\prime }\;m_{\lambda^{\prime }j}/\lambda \;m_{\lambda i}}$ is the conditional probability to find an atom of type λ′ at site (*lj*) for the atom with magnetic moment *m*_λ′*j*_, provided that the sites in the unit cell at the origin (0*i*) have an atom of type λ with a magnetic moment *m*_λ*i*_. Here, $t_{ni}^{\lambda \;m_{\lambda i}}$ is the value of the matrix element of a single-center operator for scattering in the case where an atom of type λ is located at site (*ni*) and has a magnetic moment *m*_λ*i*_.

When the system is disordered, we need to consider a random arrangement of the disordered atomic sites. Hence, in Equation (129), the probability of an atom of type λ to be at site (0*i*) is given by

(130) $$P_{0i}^{\lambda }=<c_{0i}^{\lambda }>,$$

where $c_{ni}^{\lambda }$ is a discrete binary random number taking the values of 1 or 0, depending on whether an atom of type λ is at site (*ni*) or not, respectively (36). The joint probabilities in Equations (126), (127), (129), and (130) are defined by the following:

(131) $$P_{lj\;\;0i}^{\lambda^{\prime }\;\lambda }=P_{0i}^{\lambda \;}P_{lj\;\;0i}^{\lambda^{\prime }/\lambda }=<c_{lj}^{\lambda^{\prime }}c_{0i}^{\lambda }>,\;P_{0i}^{\lambda \;m_{\lambda i}}=P_{0i}^{\lambda \;}\;P_{0i}^{\;m_{\lambda i}},P_{\;\;\;\;\;\;\;\;lj\;\;\;0i}^{\lambda^{\prime }\;m_{\lambda^{\prime }j}/\;\lambda m_{\lambda i}}=P_{\;lj\;\;\;0i}^{\lambda^{\prime }\;/\lambda }\;\;P_{\;\;lj\;\;\;0i}^{\;m_{\lambda^{\prime }j}/\;m_{\lambda i}}\;P_{lj\;\;0i}^{m_{\lambda^{\prime }j}\;m_{\lambda i}}=P_{0i}^{m_{\lambda i}}P_{lj\;\;0i}^{m_{\lambda^{\prime }j}/m_{\lambda i}}=<c_{lj}^{m_{\lambda^{\prime }j}}c_{0i}^{m_{\lambda i}}>.$$

The probabilities are determined by the interatomic pair correlations $\varepsilon_{lj\;\;0i}^{\;B\;B}$, $\varepsilon_{\;\;lj\;\;\;\;0i}^{\;\mu_{\lambda^{\prime }j}^{-}\;\mu_{\lambda i}^{-}}$ via [30]:

(132) $$P_{lj\;\;\;0i}^{\lambda^{\prime }/\lambda }=P_{lj}^{\lambda^{\prime }}+\frac{\varepsilon_{lj\;\;0i}^{B\;B}}{P_{0i}^{\lambda }}(\delta_{\lambda^{\prime }B}-\delta_{\lambda^{\prime }A})(\delta_{\lambda B}-\delta_{\lambda A}),\begin{array}{l} P_{lj\;\;\;0i}^{m_{\lambda^{\prime }j}/m_{\lambda i}}=P_{lj}^{m_{\lambda^{\prime }j}}+\frac{\varepsilon_{\;\;lj\;\;\;\;0i}^{\;\mu_{\lambda^{\prime }j}^{-}\;\mu_{\lambda i}^{-}}}{P_{0i}^{m_{\lambda i}}}(\delta_{m_{\lambda^{\prime }j},\mu_{j}^{-}}-\\ -\delta_{m_{\lambda^{\prime }j},\mu_{j}^{+}})\left(\delta_{m_{\lambda i},\mu_{i}^{-}}-\delta_{m_{\lambda i},\mu_{i}^{+}}\right) \end{array},$$

where δ is the Kronecker delta function. Note that the interatomic pair correlations also satisfy

(133) $$\varepsilon_{lj\;\;0i}^{BB}=<(c_{lj}^{B}-c_{j}^{B})(c_{0i}^{B}-c_{i}^{B})>,\;\varepsilon_{\;\;lj\;\;\;\;0i}^{\;\mu_{\lambda^{\prime }j}^{-}\;\mu_{\lambda i}^{-}}=<(c_{lj}^{\mu_{\lambda^{\prime }j}^{-}}-c_{j}^{\mu_{\lambda^{\prime }j}^{-}})(c_{0i}^{\mu_{\lambda i}^{-}}-c_{i}^{\mu_{\lambda i}^{-}})>.$$

The notations $P_{0i}^{\;m_{\lambda i}}$ and $P_{\;\;lj\;\;\;0i}^{\;m_{\lambda^{\prime }j}/\;m_{\lambda i}}$ indicate the probabilities of the static fluctuations of the magnetization.

As an example, when we have a binary alloy, consisting of two sublattices, and two types of atoms *A* and *B*, we obtain

(134) $$P_{0i}^{A}=x_{A}-\frac{\nu_{2}}{\nu }\eta_{a}$$

for the first sublattice and

(135) $$P_{0i}^{A}=x_{A}+\frac{\nu_{1}}{\nu }\eta_{a}$$

for the second sublattice, with

(136) $$P_{0i}^{B}=1-P_{0i}^{A}.$$

Here, ν = ν_1_ + ν_2_ is the total number of sublattice sites, *x_A_*, and *x_B_* = 1 − *x_A_* are the concentrations of the atomic components *A* and *B* in the alloy, and η*_a_* is the parameter that measures the long-range atomic order.

The two values $m_{\lambda i}^{}=\mu_{\lambda i}^{+}$ and $\mu_{\lambda i}^{-}$ represent the projections of the localized magnetic moment onto the *z* axis. The probability $P_{0i}^{\;m_{\lambda i}}$ is connected with the long-range magnetic parameter η*_m_* via the expressions

(137) $$P_{0i}^{\mu_{\lambda i}^{+}}=x_{\mu_{\lambda }^{+}}-\frac{\nu_{2}}{\nu }\eta_{m}$$

for sublattice 1 and

(138) $$P_{0i}^{\mu_{\lambda i}^{+}}=x_{\mu_{\lambda }^{+}}+\frac{\nu_{1}}{\nu }\eta_{m}$$

for sublattice 2, with

(139) $$P_{0i}^{\mu_{\lambda i}^{-}}=1-P_{0i}^{\mu_{\lambda i}^{+}}.$$

Here, $x_{\mu_{\lambda }^{+}}$ and $x_{\mu_{\lambda }^{-}}=1-x_{\mu_{\lambda }^{+}}$ are equal to the relative number of lattice sites with localized magnetic moment projections $\mu_{\lambda i}^{+}$ and $\mu_{\lambda i}^{-}$, respectively.

For an ideally ordered crystal, the Green’s function in Equation (97) is:

(140) $$G\left(\varepsilon \right)=\lim\tilde{G}\left(\varepsilon \right),\sigma \left(\varepsilon \right)\to 0,$$

where the Green’s function $\tilde{G}\left(\varepsilon \right)$ is given by Formulas (95) and (96). The energies of the electrons and phonons of the crystal are determined from the equations for the poles of the Green’s functions:

(141) $$\det\left\|\varepsilon \delta_{ii^{\prime }}\delta_{\gamma \gamma^{\prime }}-{\tilde{H}}_{i\gamma ,i^{\prime }\gamma^{\prime }}\left(k,\varepsilon \right)\right\|=0,$$

(142) $$\det\left\|\frac{\varepsilon^{2}}{\hbar^{2}}M_{i}\delta_{ii^{\prime }}\delta_{\alpha \alpha^{\prime }}-{\tilde{\Phi }}_{i\alpha ,i^{\prime }\alpha^{\prime }}\left(k,\varepsilon \right)\right\|=0,$$

where ${\tilde{H}}_{i\gamma ,i^{\prime }\gamma^{\prime }}\left(k,\varepsilon \right)$, ${\tilde{\Phi }}_{i\alpha ,i^{\prime }\alpha^{\prime }}\left(k,\varepsilon \right)$ are given by Formulas (111) and (114).

## 6. Free Energy

The Gibbs free energy or, in other words, the thermodynamic potential of the system, satisfies [27]:

(143) $$\Omega =-\Theta \;\ln\;\mathrm{Tr}(e^{-H/\Theta }).$$

The Hamiltonian *H* is defined in Equation (1). To perform the trace, we need to sum over all of the band states, but we also need to take into account the disorder averaging. The latter is commonly handled via a configurational average [26]. Using Formulas (50) and (144), we represent the thermodynamic potential in the form:

(144) $$\Omega =\langle \delta \Phi \rangle -\Theta \;S_{c}+\Omega_{e}^{\left(0\right)}+\Omega_{ph}^{\left(0\right)}+\Omega^{\prime },$$

where $\Omega_{e}^{\left(0\right)},\Omega_{ph}^{\left(0\right)}$ are the thermodynamic potentials for the electrons and the phonons in the field of the ionic cores, respectively. $\Omega^{\prime }$ is the component of the thermodynamic potential that is caused by the mutual scattering of electrons and phonons; it is defined by

(145) $$\Omega^{\prime }=-\Theta \;\ln\langle <\sigma (1/\Theta ){>}_{0}\rangle ,$$

with σ given in Equation (50) for the interaction picture.

In addition, $S_{c}=-<\ln P_{c}>$ is the configurational entropy, where $P_{c}$ denotes the distribution function for atoms with a specific *z*-component of the magnetic moment on a given lattice site. The angular brackets 〈…〉 denote the configurational averaging over different disorder configurations for a given density of disorder.

Next, we use the “integration over the coupling constant” method to simplify the results further. By replacing the interacting Hamiltonian $H_{\mathrm{int}}$ (defined in Equation (5)) by *H_int_*(λ) = λ*H_int_*, differentiating the expression for the piece of the thermodynamic potential $\Omega^{\prime }(\lambda )$ in Equation (146) with respect to λ and then integrating (with the boundary conditions $\Omega^{\prime }(0)=0$, $\Omega^{\prime }(1)=\Omega^{\prime }$), we obtain the following after a long derivation:

(146) Ω′=−1πνNIm∫01dλλ∫−∞∞dε[f(ε)××Tr〈(w(λ)+Σeph(ε,λ)+Σee(ε,λ))Gaa+(ε,λ)〉++12coth(ε2Θ)Tr〈ΔM−1(λ)GPP(ε,λ)++(ΔΦ(λ)+Σphph(ε,λ))Guu(ε,λ)〉].

The contribution to the thermodynamic potential from the electrons (in the field of the ionic cores) is also simple to find. It is given by

(147) $$\Omega_{e}^{\left(0\right)}=-\Theta \int_{-\infty }^{\infty }\ln\left(1+e^{(\mu_{e}-\varepsilon )/\Theta }\right)\;g_{e}^{\left(0\right)}(\varepsilon )\;d\varepsilon .$$

Similarly, the contribution to the thermodynamic potential from the phonons (in the field of the ionic cores) is given by

(148) $$\Omega_{ph}^{\left(0\right)}=\Theta \int_{-\infty }^{\infty }\ln\left(1-e^{-\varepsilon /\Theta }\right)\;g_{ph}^{\left(0\right)}(\varepsilon )\;d\varepsilon .$$

The values $g_{e}^{\left(0\right)}(\varepsilon )$ and $g_{ph}^{\left(0\right)}(\varepsilon )$ in Equations (148) and (149) are given by Formulas (126)–(130), in which one should put: $t^{\lambda m_{\lambda i}0i}=t^{\lambda 0i}=0,\tilde{G}(\varepsilon )=G_{0}(\varepsilon )$ (61)–(65).

Finally, the configurational entropy can be represented as [26]:

(149) $$S_{c}=-\left[\sum_{\lambda ,m_{\lambda i},ni}P_{ni}^{\lambda m_{\lambda i}}\ln P_{ni}^{\lambda m_{\lambda i}}+\frac{1}{2}\sum_{\begin{array}{l} \lambda ,m_{\lambda i},ni\\ \lambda^{\prime },m_{\lambda^{\prime }j},lj\\ \left(ni)\neq (lj\right) \end{array}}P_{nilj}^{\lambda m_{\lambda i}\lambda^{\prime }m_{\lambda^{\prime }j}}\ln\frac{P_{nilj}^{\lambda m_{\lambda i}\lambda^{\prime }m_{\lambda^{\prime }j}}}{P_{ni}^{\lambda m_{\lambda i}}P_{lj}^{\lambda^{\prime }m_{\lambda^{\prime }j}}}+\cdots \right]$$

Ultimately, we are interested in determining the Helmholz free energy, F, as a function of the volume V,the temperature T, the number of electrons *N_e_*, and the parameters of interatomic and magnetic correlations $\left(\varepsilon_{n_{1}i_{1}n_{2}i_{2}},\eta \right)$. The Helmholz free energy can be found directly from the thermodynamic potential. Namely, it satisfies $F=\Omega +\mu_{e}<N_{e}>$. The free energy per atom, can be approximated by [26]:

(150) $$F=\langle \delta \Phi \rangle -\Theta S_{c}+\Omega_{e}+\Omega_{ph}+\mu_{e}<Z>,$$

where $\Omega_{e}$ and $\Omega_{ph}$ are given by Equations (148) and (149), but with $g_{e}^{\left(0\right)}(\varepsilon )$, $g_{ph}^{\left(0\right)}(\varepsilon )$ replaced by $g_{e}(\varepsilon )$, $g_{ph}(\varepsilon )$ (see Equations (126)–(130)).The values of the parameters of the interatomic and magnetic correlations $\left(\varepsilon_{n_{1}i_{1}n_{2}i_{2}},\eta \right)$ are found from the condition for the minimum free energy F (151).

## 7. Electrical Conductivity

Assuming the system to be driven not too far from equilibrium, we are allowed then to make use of the linear response formalism of Kubo for the electrical conductivity tensor [33],

(151) $$\sigma_{\alpha \beta }\left(\omega \right)=\int_{0}^{1/\Theta }\int_{0}^{\infty }e^{i\omega t-\delta t}\langle {\tilde{J}}_{\beta }\left(0\right){\tilde{J}}_{\alpha }\left(t+i\hbar \tau \right)\rangle d\tau dt.$$

In this equation, $J_{\alpha }$ is the current operator along the α spatial direction. The real part of the conductivity, called the optical conductivity, can then be represented in terms of the imaginary part of the retarded response function, or equivalently as

(152) Reσαβ(ω)=i2ω[GrJαJβ(ω)−GaJαJβ(ω)],

in terms of the retarded and advanced response functions. The current operator is just the number operator for the electrons, multiplied by their velocity and the electric charge, and then summed over all states. It is compactly represented via

(153) $$J_{\alpha }\left(t\right)=e\int \Psi^{+}\left(\xi ,t\right)v_{\alpha }\Psi \left(\xi ,t\right)d\xi ,$$

where $\Psi^{+}\left(\xi ,t\right)$ and Ψ(ξ,t) are the field operators for the creation and annihilation of electrons, respectively, $\nu_{\alpha }$ is the operator of the α component of the band velocity, and e is the electron charge. The integration over ξ sums over all states.

To get the retarded response function on the real frequency axis, we must analytically continue the thermal response functions. The thermal current–current response function is defined to be

(154) $$G^{J_{\alpha }J_{\beta }}(\tau ,\tau^{\prime })=\frac{e^{2}}{NV_{1}}\sum_{n_{1}n_{2}n_{3}n_{4}}v_{\alpha n_{4}n_{2}}v_{\beta n_{3}n_{1}}G^{\prime \prime }\left(n_{1}\tau^{\prime },n_{2}\tau ,n_{3}\tau^{\prime },n_{4}\tau \right),$$

where *V*_1_ is the volume of the primitive unit cell, and the two-particle thermal Green’s function is given by the following time-ordered expectation value:

(155) G″(n1τ′,n2τ,n3τ′,n4τ)=〈Tτan1(τ′)an2(τ)an2+(τ)an4+(τ)σ(1θ)〉〈σ(1θ)〉−1,(n=niγ).

**Figure 6:**
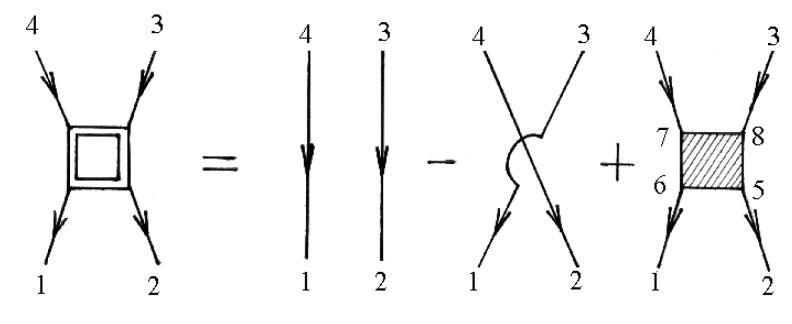
Diagrams for the two-particle Green’s function.

The two-particle Green’s function from Equation (156) is described by the diagram in Figure 6.

The numbers of Figure 6 correspond to point numbers, e.g., 1 corresponds to $(n_{1}i_{1}\gamma_{1}\tau_{1}).$

Using the diagram technique for two-particle temperature Green’s function and neglecting the contributions of scattering processes on clusters of three or more sites for the conductivity tensor, we can get:

(156) Reσαβ(ω)=e2ℏ4πV1ε{∫−∞∞dε1[f(ε1+ε)−f(ε1)]∑s,s′=+,−(2δss′−1)∑γ,i{[vβK˜(ε1s,vα,ε1s′+ε)]+∑λ,mλiP0iλmλiK˜(ε1s′+ε,vβ,ε1s)(tλmλi0i(ε1s)K˜(ε1s,vα,ε1s′+ε)tλmλi0i(ε1s′+ε)+∑λ,mλiP0iλmλi∑lj≠0i,λ′,mλ′jPlj   0iλ′mλ′j/λmλi[[K˜(ε1s′+ε,vβ,ε1s)vαG˜(ε1s′+ε)]×T(2)λmλi0i,λ′mλ′jlj(ε1s′+ε)+[K˜(ε1s,vα,ε1s′+ε)vβG˜(ε1s)]T(2)λ mλi0i,λ′mλ′jlj(ε1s)+K˜(ε1s′+ε,vβ,ε1s)[(tλ′mλ′jlj(ε1s)K˜(ε1s,vα,ε1s′+ε)tλmλi0i(ε1s′+ε)+(tλmλi0i(ε1s)+tλ′mλ′jlj(ε1s))K˜(ε1s,vα,ε1s′+ε)T(2)λ mλi0i,λ′mλ′jlj(ε1s′+ε)+T(2)λ′mλ′jlj,λmλi0i(ε1s)K˜(ε1s,vα,ε1s′+ε)tλmλi0i(ε1s′+ε)+T(2)λ′mλ′jlj,λmλi0i(ε1s)K˜(ε1s,vα,ε1s′+ε)T(2)λmλi0i,λ′mλ′jlj(ε1s′+ε)+T(2)λ′mλ′jlj,λmλi0i(ε1s)K˜(ε1s,vα,ε1s′+ε)T(2)λ′mλ′jlj,λmλi0i(ε1s′+ε)]]}0iγ,0iγ+1N∫−∞∞∫−∞∞dε1dε2f(ε1)f(ε2)ΔGαβII(ε1,ε2;ε)},

where

(157) $$\tilde{K}\left(\varepsilon_{1}^{s},v_{\alpha },\varepsilon_{1}^{s^{\prime }}+\varepsilon \right)={\tilde{G}}^{aa^{+}}\left(\varepsilon_{1}^{s}\right)v_{\alpha }{\tilde{G}}^{aa^{+}}\left(\varepsilon_{1}^{s^{\prime }}+\varepsilon \right),\;{\tilde{G}}^{aa^{+}}\left(\varepsilon_{1}^{+}\right)={\tilde{G}}_{r}^{aa^{+}}\left(\varepsilon_{1}\right),\;{\tilde{G}}^{aa^{+}}\left(\varepsilon_{1}^{-}\right)={\tilde{G}}_{a}^{aa^{+}}\left(\varepsilon_{1}\right)={\left({\tilde{G}}_{r}^{aa^{+}}\right)}^{*}\left(\varepsilon_{1}\right),$$

And the two-particle interaction term denoted by $\Delta G^{II}(\varepsilon_{1},\varepsilon_{2}),$ is given by the equation:

(158) $$\Delta G_{\alpha \beta }^{II}\left(\varepsilon_{1},\varepsilon_{2};\varepsilon \right)=\frac{i}{2\pi }v_{\alpha n_{4}n_{2}}v_{\beta n_{3}n_{1}}\{[{\tilde{G}}_{rn_{1}n_{6}}^{aa^{+}}\left(\varepsilon_{1}\right)-{\tilde{G}}_{an_{1}n_{6}}^{aa^{+}}\left(\varepsilon_{1}\right)]\times \;\times [{\tilde{G}}_{rn_{2}n_{5}}^{aa^{+}}\left(\varepsilon_{2}\right)-{\tilde{G}}_{an_{2}n_{5}}^{aa^{+}}\left(\varepsilon_{2}\right)][{\tilde{G}}_{an_{7}n_{4}}^{aa^{+}}\left(\varepsilon_{2}-\varepsilon \right){\tilde{G}}_{rn_{8}n_{3}}^{aa^{+}}\left(\varepsilon_{1}+\varepsilon \right)-\;-{\tilde{G}}_{rn_{7}n_{4}}^{aa^{+}}\left(\varepsilon_{2}-\varepsilon \right){\tilde{G}}_{an_{8}n_{3}}^{aa^{+}}\left(\varepsilon_{1}+\varepsilon \right)]+{\tilde{G}}_{an_{1}n_{6}}^{aa^{+}}\left(\varepsilon_{1}-\varepsilon \right)[{\tilde{G}}_{rn_{2}n_{5}}^{aa^{+}}\left(\varepsilon_{2}\right)-{\tilde{G}}_{an_{2}n_{5}}^{aa^{+}}\left(\varepsilon_{2}\right)]\times \;\times {\tilde{G}}_{an_{7}n_{4}}^{aa^{+}}\left(\varepsilon_{2}-\varepsilon \right)[{\tilde{G}}_{rn_{8}n_{3}}^{aa^{+}}\left(\varepsilon_{1}\right)-{\tilde{G}}_{an_{8}n_{3}}^{aa^{+}}\left(\varepsilon_{1}\right)]-{\tilde{G}}_{rn_{1}n_{6}}^{aa^{+}}\left(\varepsilon_{1}-\varepsilon \right)\times \;\times [{\tilde{G}}_{rn_{2}n_{5}}^{aa^{+}}\left(\varepsilon_{2}\right)-{\tilde{G}}_{an_{2}n_{5}}^{aa^{+}}\left(\varepsilon_{2}\right)]{\tilde{G}}_{rn_{7}n_{4}}^{aa^{+}}\left(\varepsilon_{2}-\varepsilon \right)[{\tilde{G}}_{rn_{8}n_{3}}^{aa^{+}}\left(\varepsilon_{1}\right)-{\tilde{G}}_{an_{8}n_{3}}^{aa^{+}}\left(\varepsilon_{1}\right)]+\;+\left[{\tilde{G}}_{an_{1}n_{6}}^{aa^{+}}\left(\varepsilon_{1}-\varepsilon \right){\tilde{G}}_{rn_{2}n_{5}}^{aa^{+}}\left(\varepsilon_{2}+\varepsilon \right)-{\tilde{G}}_{rn_{1}n_{6}}^{aa^{+}}\left(\varepsilon_{1}-\varepsilon \right){\tilde{G}}_{an_{2}n_{5}}^{aa^{+}}\left(\varepsilon_{2}+\varepsilon \right)\right]\times \;\times [{\tilde{G}}_{rn_{7}n_{4}}^{aa^{+}}\left(\varepsilon_{2}\right)-{\tilde{G}}_{an_{7}n_{4}}^{aa^{+}}\left(\varepsilon_{2}\right)][{\tilde{G}}_{rn_{8}n_{3}}^{aa^{+}}\left(\varepsilon_{1}\right)-{\tilde{G}}_{an_{8}n_{3}}^{aa^{+}}\left(\varepsilon_{1}\right)]+\;+[{\tilde{G}}_{rn_{1}n_{6}}^{aa^{+}}\left(\varepsilon_{1}\right)-{\tilde{G}}_{an_{1}n_{6}}^{aa^{+}}\left(\varepsilon_{1}\right)]{\tilde{G}}_{rn_{2}n_{5}}^{aa^{+}}\left(\varepsilon_{2}+\varepsilon \right)[{\tilde{G}}_{rn_{7}n_{4}}^{aa^{+}}\left(\varepsilon_{2}\right)-{\tilde{G}}_{an_{7}n_{4}}^{aa^{+}}\left(\varepsilon_{2}\right)]\;\times {\tilde{G}}_{rn_{8}n_{3}}^{aa^{+}}\left(\varepsilon_{1}+\varepsilon \right)-[{\tilde{G}}_{rn_{1}n_{6}}^{aa^{+}}\left(\varepsilon_{1}\right)-{\tilde{G}}_{an_{1}n_{6}}^{aa^{+}}\left(\varepsilon_{1}\right)]{\tilde{G}}_{an_{2}n_{5}}^{aa^{+}}\left(\varepsilon_{2}+\varepsilon \right)\times \;\times [{\tilde{G}}_{rn_{7}n_{4}}^{aa^{+}}\left(\varepsilon_{2}\right)-{\tilde{G}}_{an_{7}n_{4}}^{aa^{+}}\left(\varepsilon_{2}\right)]{\tilde{G}}_{an_{8}n_{3}}^{aa^{+}}\left(\varepsilon_{1}+\varepsilon \right)\}{\tilde{\Gamma }}_{n_{5}n_{8}}^{n_{6}n_{7}},\;\left(n=ni\gamma \right)$$

Summation over repeated indices in expression (159) is implied.

For the static conductivity tensor, we can get (ω→0):

(159) $$\begin{array}{l} \sigma_{\alpha \beta }=\frac{e^{2}\hbar }{4\pi V_{1}}\{\int_{-\infty }^{\infty }d\varepsilon_{1}\frac{\partial f}{\partial \varepsilon_{1}}\sum_{s,s^{\prime }=+,-}\left(2\delta_{ss^{\prime }}-1\right)\sum_{\gamma ,i}\{\left[v_{\beta }\tilde{K}\left(\varepsilon_{1}^{s},v_{\alpha },\varepsilon_{1}^{s^{\prime }}\right)\right]\\ +\sum_{\lambda ,m_{\lambda i}}P_{0i}^{\lambda m_{\lambda i}}\tilde{K}(\varepsilon_{1}{}^{s^{\prime }},v_{\beta },\varepsilon_{1}{}^{s})(t^{\lambda m_{\lambda i}0i}(\varepsilon_{1}{}^{s})\tilde{K}(\varepsilon_{1}{}^{s},v_{\alpha },\varepsilon_{1}{}^{s^{\prime }})t^{\lambda m_{\lambda i}0i}(\varepsilon_{1}{}^{s^{\prime }})\\ +\sum_{\lambda ,m_{\lambda i}}P_{0i}^{\lambda m_{\lambda i}}\sum_{\begin{array}{l} lj\neq 0i,\\ \lambda^{\prime },m_{\lambda^{\prime }j} \end{array}}P_{lj\;\;\;0i}^{\lambda^{\prime }m_{\lambda^{\prime }j}/\lambda m_{\lambda i}}[\left[\tilde{K}(\varepsilon_{1}{}^{s^{\prime }},v_{\beta },\varepsilon_{1}{}^{s})v_{\alpha }\tilde{G}(\varepsilon_{1}{}^{s^{\prime }}\right)]\\ \times T^{(2)\lambda m_{\lambda i}0i,\lambda^{\prime }m_{\lambda^{\prime }j}lj}(\varepsilon_{1}{}^{s^{\prime }})\\ +[\tilde{K}(\varepsilon_{1}{}^{s},v_{\alpha },\varepsilon_{1}{}^{s^{\prime }})v_{\beta }\tilde{G}(\varepsilon_{1}{}^{s})]T^{(2)\lambda \;m_{\lambda i}0i,\lambda^{\prime }m_{\lambda^{\prime }j}lj}(\varepsilon_{1}{}^{s})\\ +\tilde{K}(\varepsilon_{1}{}^{s^{\prime }},v_{\beta },\varepsilon_{1}{}^{s})[\left(t^{\lambda^{\prime }m_{\lambda^{\prime }j}lj}(\varepsilon_{1}{}^{s})\tilde{K}(\varepsilon_{1}{}^{s},v_{\alpha },\varepsilon_{1}{}^{s^{\prime }})t^{\lambda m_{\lambda i}0i}(\varepsilon_{1}{}^{s^{\prime }}\right)\\ +\left(t^{\lambda m_{\lambda i}0i}(\varepsilon_{1}{}^{s})+t^{\lambda^{\prime }m_{\lambda^{\prime }j}lj}(\varepsilon_{1}{}^{s})\right)\tilde{K}(\varepsilon_{1}{}^{s},v_{\alpha },\varepsilon_{1}{}^{s^{\prime }})T^{(2)\lambda \;m_{\lambda i}0i,\lambda^{\prime }m_{\lambda^{\prime }j}lj}(\varepsilon_{1}{}^{s^{\prime }})\\ +T^{(2)\lambda^{\prime }m_{\lambda^{\prime }j}lj,\lambda m_{\lambda i}0i}(\varepsilon_{1}{}^{s})\tilde{K}(\varepsilon_{1}{}^{s},v_{\alpha },\varepsilon_{1}{}^{s^{\prime }})t^{\lambda m_{\lambda i}0i}(\varepsilon_{1}{}^{s^{\prime }})\\ +T^{(2)\lambda^{\prime }m_{\lambda^{\prime }j}lj,\lambda m_{\lambda i}0i}(\varepsilon_{1}{}^{s})\tilde{K}(\varepsilon_{1}{}^{s},v_{\alpha },\varepsilon_{1}{}^{s^{\prime }})T^{(2)\lambda m_{\lambda i}0i,\lambda^{\prime }m_{\lambda^{\prime }j}lj}(\varepsilon_{1}{}^{s^{\prime }})\\ +{T^{(2)\lambda^{\prime }m_{\lambda^{\prime }j}lj,\lambda m_{\lambda i}0i}(\varepsilon_{1}{}^{s})\tilde{K}(\varepsilon_{1}{}^{s},v_{\alpha },\varepsilon_{1}{}^{s^{\prime }})T^{(2)\lambda^{\prime }m_{\lambda^{\prime }j}lj,\lambda m_{\lambda i}0i}(\varepsilon_{1}{}^{s^{\prime }})]]\}}^{0i\gamma ,0i\gamma }\} \end{array}$$

The electron velocity satisfies the conventional definition

(160) $$v_{\alpha }\left(k\right)=\frac{1}{\hbar }\frac{\partial H_{0}^{\left(1\right)}\left(k\right)}{\partial k_{\alpha }}.$$

When deriving expression (160), the last small term in the expression for electrical conductivity (157) is neglected.

The method developed in this work was applied in [28] to study the effect of an impurity on the energy spectrum and electrical conductivity of carbon nanotubes.

## 8. Energy Spectrum of Graphene with Adsorbed Potassium Atoms

To calculate the electron spectrum of graphene with adsorbed potassium atoms, we chose the wave functions of the *2s* and *2p* states of neutral noninteracting carbon atoms as the basis. In the calculation of the matrix elements of the Hamiltonian, we took three first coordination spheres. The energy spectrum of graphene was calculated for the temperature *T* = 0 K. In calculations, we neglect the renormalization of vertices of the mass operator of the electron–electron interaction. The dependence of the energy of an electron on the wave vector for graphene is calculated from the equation for Green’s function poles for the electron subsystem, defined in Equation (142).

**Figure 7:**
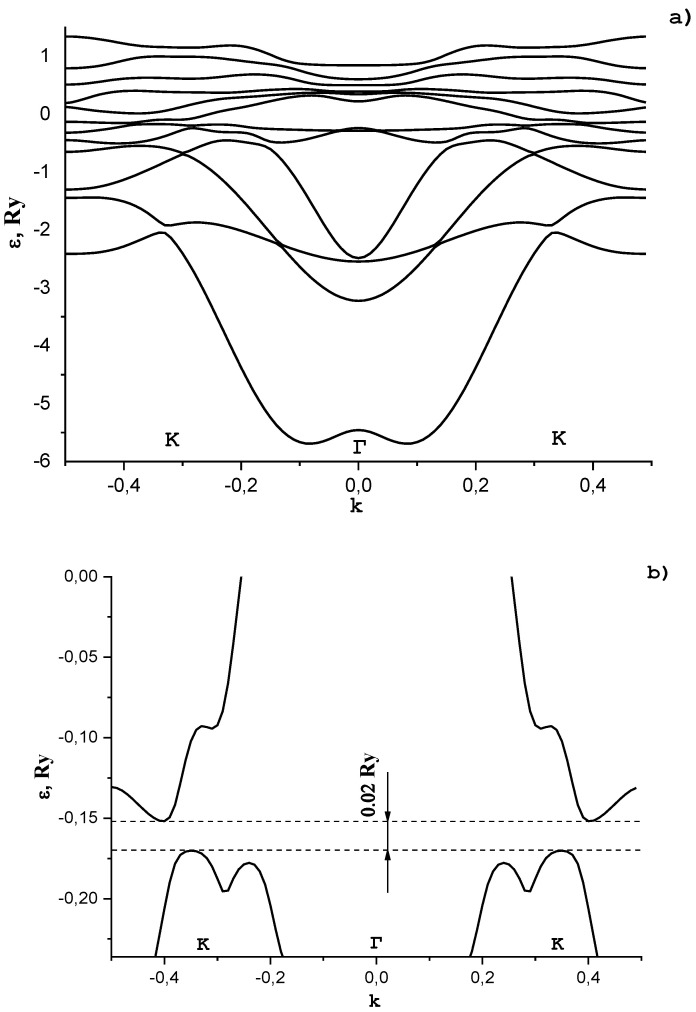
Electronic spectrum of graphene with impurities. The dependence of the energy ε on the wave vector k in the region of the slit is shown in (**a**). (**b**) gap in the energy spectrum of graphene arises.

In Figure 7a, we show the dependence of the electron energy ε in graphene with adsorbed potassium atoms on the wave vector **k**. Vector **k** is directed from the Brillouin zone center (point Γ) to the Dirac point (point K).

In Figure 7, the structural periodic distance from a potassium atom to a carbon atom is 0.28 nm. It is seen from Figure 7 that, at the ordered arrangement of potassium atoms, a gap in the energy spectrum of graphene arises. Its value depends on the concentration of adsorbed potassium atoms, their location in the unit cell, and the distance to carbon atoms. We established that, at the potassium concentration such that the unit cell includes two carbon atoms and one potassium atom, the latter being placed on the graphene surface above a carbon atom at a distance of 0.286 nm, the energy gap is ~0.25 eV (see Figure 7b). A more complex dependence of the electron energy on the wave vector in the region of the energy gap in comparison with that previously investigated in [34,35,36] in a simple two-band model is due to the effect of band hybridization. The location of the Fermi level in the energy spectrum depends on the potassium concentration and is in the energy interval $-0.36\;\mathrm{Ry}\leq \varepsilon_{F}\leq Ry0.36$. Such a situation is realized if graphene is placed on a potassium support.

## 9. Conclusions

A novel approach to the description of the electronic spectrum, the thermodynamic potential, and the electrical conductivity of disordered crystals, based on the Hamiltonian of electrons and phonons, constitutes the main issue of the present work. Expressions for Green’s functions, thermodynamic potential, and electrical conductivity are derived using the diagram method. Equations are obtained for the vertex parts of the mass operators of electron–electron and electron–phonon interactions. A system of exact equations is obtained for the spectrum of elementary excitations in a crystal. This makes it possible to perform numerical calculations of the energy spectrum and the properties of the system with a predetermined accuracy. In contrast to other approaches, in which electron correlations are taken into account only in the limiting cases of an infinitely large and infinitesimal electron density, in this method, electron correlations are described in the general case of an arbitrary density.

It was found that a gap appears in the energy spectrum of graphene with an ordered arrangement of potassium atoms. Its value depends on the concentration of adsorbed potassium atoms, their location in the unit cell, and the distance to carbon atoms. It was found that at such a concentration of potassium, the unit cell includes two carbon atoms and one potassium atom, the latter being located on the graphene surface above the carbon atom at a distance of 0.286 nm, and the band gap is ~0.25 eV. Such a situation is realized if graphene is placed on a potassium support. A more complex dependence of the electron energy on the wave vector in the region of the energy gap in comparison with that previously investigated in [34,35,36] in a simple two-band model is due to the effect of band hybridization.

## Data Availability

Data are available by the Corresponding Author upon reasonable request.
